# Using sulfur bridge oxidation to control electronic coupling and photochemistry in covalent anthracene dimers†
†Electronic supplementary information (ESI) available. See DOI: 10.1039/c8sc05598j


**DOI:** 10.1039/c8sc05598j

**Published:** 2019-06-17

**Authors:** Chad D. Cruz, Jennifer Yuan, Clàudia Climent, Nathan T. Tierce, Peter R. Christensen, Eric L. Chronister, David Casanova, Michael O. Wolf, Christopher J. Bardeen

**Affiliations:** a Department of Chemistry , University of California Riverside , 501 Big Springs Road, Riverside , California 92521 , USA . Email: christopher.bardeen@ucr.edu; b Department of Chemistry , University of British Columbia , 2036 Main Mall , Vancouver , BC , Canada V6T 1Z1; c Departamento de Física Teórica de la Materia Condensada , Universidad Autónoma de Madrid , E-28049 Madrid , Spain; d Donostia International Physics Center (DIPC) , Paseo Manuel de Lardizabal 4 , 20018 Donostia , Euskadi , Spain; e IKERBASQUE , Basque Foundation for Science , 48013 Bilbao , Euskadi , Spain

## Abstract

Covalently tethered bichromophores provide an ideal proving ground to develop strategies for controlling excited state behavior in chromophore assemblies. In this work, optical spectroscopy and electronic structure theory are combined to demonstrate that the oxidation state of a sulfur linker between anthracene chromophores gives control over not only the photophysics but also the photochemistry of the molecules. Altering the oxidation state of the sulfur linker does not change the geometry between chromophores, allowing electronic effects between chromophores to be isolated. Previously, we showed that excitonic states in sulfur-bridged terthiophene dimers were modulated by electronic screening of the sulfur lone pairs, but that the sulfur orbitals were not directly involved in these states. In the bridged anthracene dimers that are the subject of the current paper, the atomic orbitals of the unoxidized S linker can actively mix with the anthracene molecular orbitals to form new electronic states with enhanced charge transfer character, different excitonic coupling, and rapid (sub-nanosecond) intersystem crossing that depends on solvent polarity. However, the fully oxidized SO_2_ bridge restores purely through-space electronic coupling between anthracene chromophores and inhibits intersystem crossing. Photoexcitation leads to either internal conversion on a sub-20 picosecond timescale, or to the creation of a long-lived emissive state that is the likely precursor of the intramolecular [4 + 4] photodimerization. These results illustrate how chemical modification of a single atom in the covalent bridge can dramatically alter not only the photophysics but also the photochemistry of molecules.

## Abstract 2

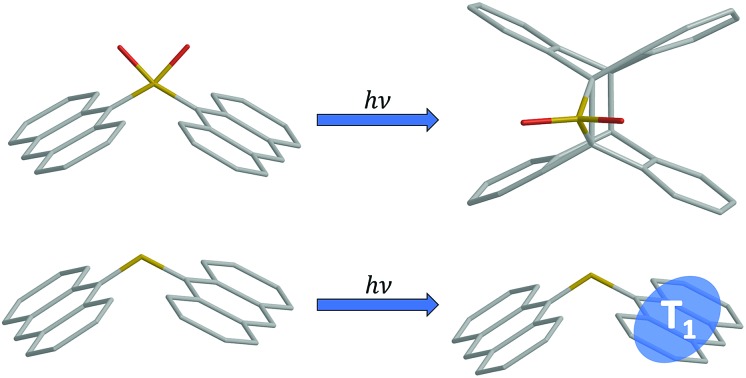
For anthracene dimers bridged by a sulfur atom, modulating the sulfur oxidation state profoundly affects excited state behavior. The SO_2_-bridge supports long-lived states and photodimerization, while the S-bridge undergoes intersystem crossing.

## 1. Introduction

The application of conjugated organic molecules in solar energy conversion and electroluminescence requires the development of complex chromophores and control of their assembly. The geometric arrangement of molecular chromophores determines their ability to interact electronically and generate various types of exciton and charge transfer (CT) states.^1^–^5^ One way to control the interaction between chromophore molecules is to tether two of them together using a covalent linker group. This structural motif allows the geometrical arrangement between the chromophores to be defined and the electronic interaction to be controlled. Such dimers or bichromophores form the smallest subunit of larger chromophore assemblies such as polymers and are useful for basic studies of phenomena relevant to organic electronic materials. For example, bichromophores have been used to study energy and charge transfer,^6^–^14^ as well as singlet exciton fission^15^–^25^ and triplet–triplet annihilation.^26^,^27^ They can also support intramolecular CT states that give rise to enhanced charge separation and thermally activated delayed fluorescence.^28^–^30^ Their unique properties may eventually lead to applications in technologies including light-emitting diodes and photovoltaics.

One of the most important scientific questions regarding covalent bichromophores concerns the role of the linker. In one limit, it functions as a passive structural element that positions the two chromophores to interact purely *via* through-space Coulombic effects. But in many cases, it can actively participate in the electronic coupling through its own electronic states.^31^–^34^ Separating these two effects can be challenging because alterations to the linker's chemical structure to modify its electronic properties typically changes the molecular geometry and the through-space interaction simultaneously. Wolf and coworkers developed a way around this problem by using a sulfur atom as a bichromophore bridge.^35^,^36^ Changing the oxidation state of the sulfur from S to SO to SO_2_ allows modification of the electronic properties of the bridge while maintaining almost identical molecular conformations, as determined from the crystal structures. Modification of the electronic structure of the bridge resulted in dramatic changes in the photophysical properties of bichromophores based on pyrene, naphthalene and terthiophene. In the case of terthiophene dimers, oxidation of the sulfur bridge turned off the intersystem crossing (ISC) that normally limits the fluorescence yields of monomeric terthiophene.^14^ When the bridge was fully oxidized, both experimental and computational results indicated that an excited state with appreciable CT character could be formed in the dimers. This low-lying state limits ISC and generated fluorescence with a quantum yield of 52% in polar solvents, as compared to 6% for monomeric terthiophene. The main physical insight that emerged from that work was that the electron pairs on the bridging sulfur atom could be used to modulate the chromophore–chromophore interaction. The presence of the lone pair electrons between the two molecular π systems hinders π–π interactions and suppresses intramolecular CT states. Replacing the lone pairs with polarized S=O bonds prevents them from screening out the Coulombic stabilization of intramolecular CT states, enabling the formation of longer-lived emissive excited states.

**Chart 1:**
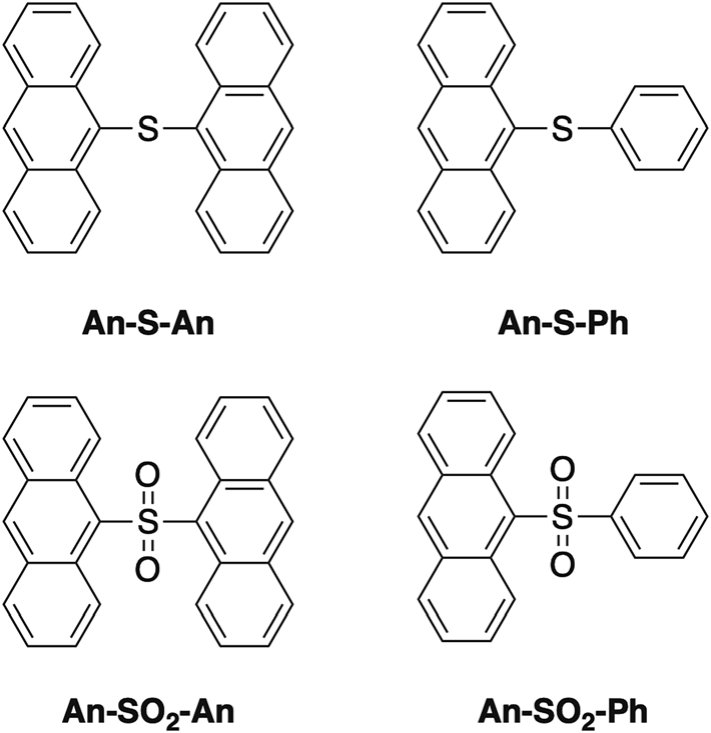
Molecular structures of compounds in this study.

We were interested to see if the principles derived from the terthiophene work can be extended to dimeric systems composed of other chromophores. We chose anthracene (**An**) because it is a conjugated organic molecule whose photophysics and photochemistry have been extensively explored.^37^–^39^ Unlike terthiophene, it does not possess an intrinsic heavy atom like sulfur, so ISC is weaker in the monomeric form. Moreover, **An** can undergo photochemical reactions in solution, specifically [4 + 4] photocycloaddition between neighboring **An** rings. This capability opens up the possibility of using the linker group to modify the photoreactivity of the bichromophore as well. The **An**-based bichromophores used in this study are shown in Chart 1. The current study was motivated by our preliminary study of the **An–SO*_x_*–An** (*x* = 0, 1, 2) linked dimers, where it was shown that their photoreactivity depended on the linker oxidation state.^36^ Under UV excitation **An–SO_2_–An** formed a photodimer containing an episulfone ring whereas **An–S–An** was unreactive.^36^ We note that **An–SO–An** was explored in the previous study as well and was found to have unique photochemistry in which the bridging sulfoxide was lost and 9,9′-bianthryl was formed. We did not include the sulfoxide linker in the current work because (1) the efficient and irreversible photochemistry makes obtaining reliable photophysical data challenging; and (2) we wanted to concentrate on the limiting cases of S (not oxidized) *versus* SO_2_ (fully oxidized).

Electron donating and withdrawing substituents have been widely used to modify the photophysics of **An** derivatives.^40^–^46^ In most cases, the substituent can undergo an electron transfer reaction with the photoexcited **An** core, leading to the formation of the **An** radical cation or anion. Subsequent charge recombination can lead to triplet formation and interesting photochemistry.^47^,^48^ Here, we are interested in a subtler effect: the use of the linker atom to modulate the excited state structure *via* CT interactions while avoiding full electron transfer. The ability to generate novel excited states without ionization can be expected to lead to new photophysical behavior. In this paper, we combine organic synthesis, spectroscopy and theory to understand the origin of the differences in reactivity for the **An**-based bichromophores. Dimers with different bridge oxidation states (S *versus* SO_2_) and constituent chromophores (**An***versus* phenyl (**Ph**)) are prepared in order to isolate bridge and chromophore effects. Steady-state and time-resolved spectroscopy measurements are used to characterize the excited state dynamics, while time-dependent density functional theory (TD-DFT) calculations are used to characterize the nature of the electronic states. At the start of this study, we expected that the role of the oxidation state of the bridging S atom would be similar to that in the terthiophene bichromophores, where the bridge lone pair orbitals modulate the electronic interactions between the **An** chromophores without participating in the electronic states. However, we find that this separation does not hold for the S-bridge, whose orbitals do participate in the optically accessible excited states and contribute to rapid ISC in both **An–S–An** and **An–S–Ph** bichromophores. The SO_2_-bridged dimers exhibit more complex dynamics, with rapid internal conversion and, in the **An–SO_2_–An** molecules, the generation of a long-lived emissive state that is likely the precursor to the intramolecular [4 + 4] photocycloaddition. Intramolecular reactivity in covalent anthracene assemblies is usually assumed to be completely controlled by steric effects and geometry, for example through the topochemical principle.^49^–^53^ Our results suggest that electronic state engineering, which can be accomplished by changing the chemical structure of a single atom in the assembly, provides a new chemical strategy for controlling the photochemical behavior of covalent molecular assemblies.

## 2. Experimental

The symmetric sulfur-bridged anthracene dimers **An–S–An** and **An–SO_2_–An** are synthesized in accordance with a previously reported method.^36^ The synthetic scheme to make **An–S–Ph** and **An–SO_2_–Ph** is given in Scheme S1 and described in detail in the ESI.†

Steady-state absorption measurements of each compound in dilute solutions of cyclohexane (∼30 μM), CH_2_Cl_2_ (∼100 μM), and acetonitrile (∼50 μM) are performed with a Varian Cary 500 spectrophotometer. Steady-state photoluminescence (PL) spectra of the same solutions are recorded in a right-angle configuration with a PTI QM-400 fluorimeter.

Time-resolved PL experiments are performed by frequency doubling the 800 nm output of a 1 kHz repetition rate Coherent Libra Ti:sapphire laser system. The PL signal is detected using a Hamamatsu C4334 Streakscope which has a time resolution of 25 ps and a wavelength resolution of 2.5 nm. The sample solutions are contained in a 1 cm quartz cuvette and the emitted light is collected in a front-face configuration utilizing magic angle polarization. A 420 nm long wave pass filter is placed before the detector to minimize laser scatter in the signal. Each solution is degassed by bubbling argon gas through the cuvette for 15 minutes prior to the measurement.

Femtosecond transient absorption (TA) measurements are performed using the 1 kHz laser system described above. The 400 nm beam is used as the pump and a small portion of the 800 nm fundamental beam is focused onto a CaF_2_ plate to generate the white light continuum probe beam. The pump and probe beams are overlapped in a 1 mm pathlength quartz flow cell. The probe beam is detected with a fiber optic coupled to an Ocean Optics S2000 spectrometer. The Ultrafast Systems Helios program is used to control the delay stage as well as generate the difference spectrum. The solvent response is also measured and nonresonant contributions to the TA signal are removed with Ultrafast Systems Surface Xplorer software. Pump-pulse fluences are kept between 0.6–3 mJ cm^–2^. The time resolution of these experiments is estimated to be ∼150 fs based on the width of the pump-probe cross correlation signal from the neat solvent.

Electronic structure calculations for the ground and excited states are performed within the framework of density functional theory (DFT)^54^,^55^ and its time-dependent version (TD-DFT)^56^,^57^ respectively. To account for weak interactions and important electronic redistribution between the anthracene moieties and the SO_*x*_ bridge upon photoexcitation, the ωB97X-D functional^58^ is used together with the 6-31+G(d) basis set. CH_2_Cl_2_ solvent effects are accounted for with the polarizable continuum model using the C-PCM variant.^59^ Critical points on the ground state potential energy surface (PES) are optimized with no restrictions. Computation of the diabatic states is performed by means of the Edmiston–Ruedenberg localization scheme.^60^ Energy crossing points are optimized within the spin-flip DFT (SF-DFT) approximation^61^ with the BHHLYP functional.^62^,^63^ All calculations are performed with the Q-Chem program.^64^

## 3. Results and discussion

### 3.1 Steady-state spectroscopy

**Fig. 1:**
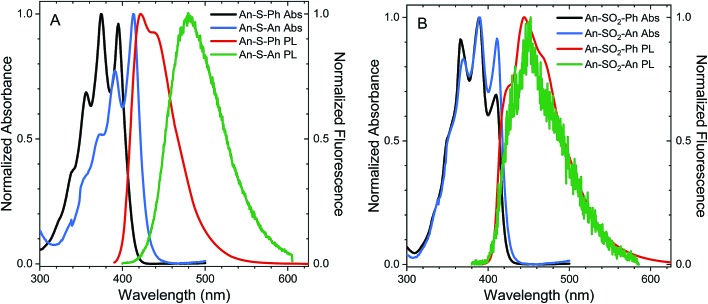
The steady state absorption and PL spectra of the S-bridged (A) and SO_2_-bridged (B) monomers in dilute solutions of cyclohexane. The PL spectra shown for the **An–SO*_x_*–An** dimers are from the time-resolved measurements. All PL spectra were recorded after 400 nm excitation.

The steady state absorption spectra of **An–S–An**, **An–S–Ph**, **An–SO_2_–An** and **An–SO_2_–Ph** in cyclohexane are shown in Fig. 1. The absorption spectra of all sulfur-bridged anthracenes display vibronic structure which is reminiscent of unsubstituted **An**. The enhanced relative intensity of the 0–0 vibronic peaks in both the **An**-terminated dimers indicates the presence of excitonic interaction between chromophores.^4^ This enhancement of the 0–0 peak is more pronounced for **An–S–An** than for **An–SO_2_–An**, which also undergoes a 20 nm redshift that is not observed for the oxidized species. The absorption redshift and 0–0 peak enhancement is indicative of J-type excitonic coupling in **An–S–An**. For all compounds, the absorption spectra are relatively insensitive to solvent polarity, with acetonitrile and CH_2_Cl_2_ causing only a slight broadening of the spectra (ESI, Fig. S4†).

The steady-state photoluminescence (PL) of the **Ph**-terminated compounds is measured in cyclohexane, CH_2_Cl_2_ and acetonitrile after 400 nm excitation. The PL spectra for the **An–SO*_x_*–An** compounds are extracted from the time-resolved measurements in order to avoid a long-lived impurity emission that contaminated the steady-state spectra reported in our earlier work.^36^ The PL spectrum of **An–S–Ph** peaks at 420 nm in cyclohexane with a small shoulder present at 440 nm (Fig. 1A). In solutions of CH_2_Cl_2_ and acetonitrile, the spectra are featureless and red-shift slightly ∼20 nm (ESI, Fig. S5†). When the **Ph** group is replaced by **An**, the PL of **An–S–An** in cyclohexane has a strong, featureless PL centered at 475 nm (Fig. 1A). This feature red-shifts to ∼540 nm and ∼560 nm in CH_2_Cl_2_ and acetonitrile respectively (ESI, Fig. S5†), suggesting that the emissive state possesses significant CT character. The PL spectrum of **An–SO_2_–Ph** in cyclohexane is similar to that of **An–S–Ph** (Fig. 1B), again broadening in polar solvents and shifting to lower energies (ESI, Fig. S5†). However, when the **Ph** group is replaced by **An**, the high sensitivity to the second chromophore and solvent polarity is not observed. Unlike the S-bridge, the PL spectra of **An–SO_2_–An** is similar to that of **An–SO_2_–Ph** and barely shifts with solvent polarity, although a slight broadening of the spectra is observed in CH_2_Cl_2_ and acetonitrile (ESI, Fig. S5†). The spectra retain much of the structure observed in the cyclohexane spectrum and the broadened red portions seen in the polar solvents are likely due to a species with some CT character in the excited state as they resemble excimer PL observed in methyl bridged anthracene dimers.^65^

The different behaviors observed in the steady-state spectra suggest that there are significant differences between the S- and SO_2_-bridged compounds. These differences become even more pronounced when the time-resolved data is analyzed. For this reason, in the following sections we analyze the dynamic behavior of the S- and SO_2_-bridged compounds separately before comparing them in the theoretical section of this paper.

### 3.2 Excited state dynamics of **An–S–Ph** and **An–S–An**

**Fig. 2:**
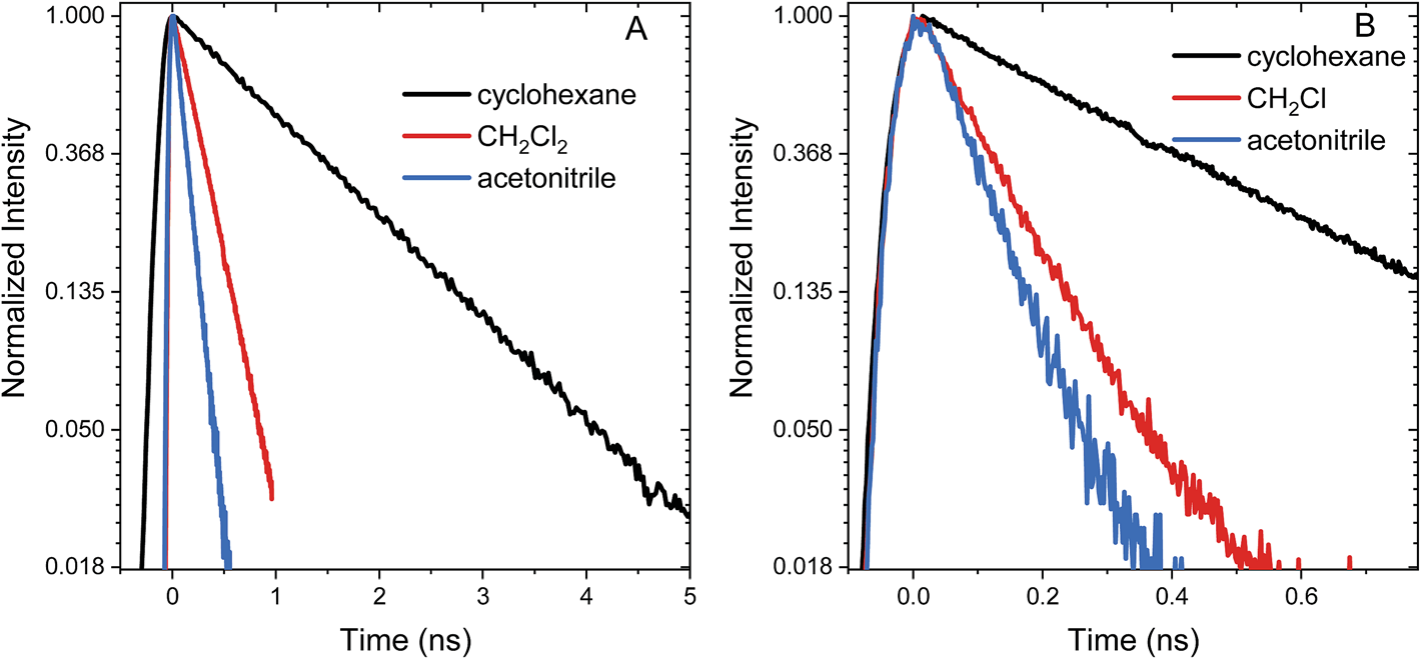
Normalized PL time traces on a natural log scale for **An–S–Ph** (A) and **An–S–An** (B) in cyclohexane, CH_2_Cl_2_, and acetonitrile. These figures show that the PL lifetime decreases with increasing solvent polarity for both **An–S–Ph** and **An–S–An**, and the second anthracene chromophore induces a more rapid decay than the phenyl terminated molecules.

Excited state relaxation in unsubstituted **An** is caused by a combination of ISC, internal conversion, and radiative relaxation.^43^ ISC in anthracene is efficient (∼70% triplet quantum yield) and contributes to a relatively short PL lifetime of ∼4 ns.^43^ The addition of the **S–Ph** moiety leads to shorter PL lifetimes of 1.4 ns in cyclohexane, 300 ps in CH_2_Cl_2_ and 130 ps in acetonitrile (Fig. 2A). Replacing the **Ph** by **An** quenches the PL more effectively with PL lifetimes of 400 ps in cyclohexane, 120 ps in CH_2_Cl_2_ and 90 ps in acetonitrile (Fig. 2B). The decays are mono-exponential at earlier times, with a slight deviation at later times caused by residual fluorescent impurities (ESI, Fig. S6†). For both S-bridged dimers, the solvent-dependent PL lifetimes suggest that an excited state with some CT character undergoes much more rapid nonradiative relaxation than monomeric **An**.

**Fig. 3:**
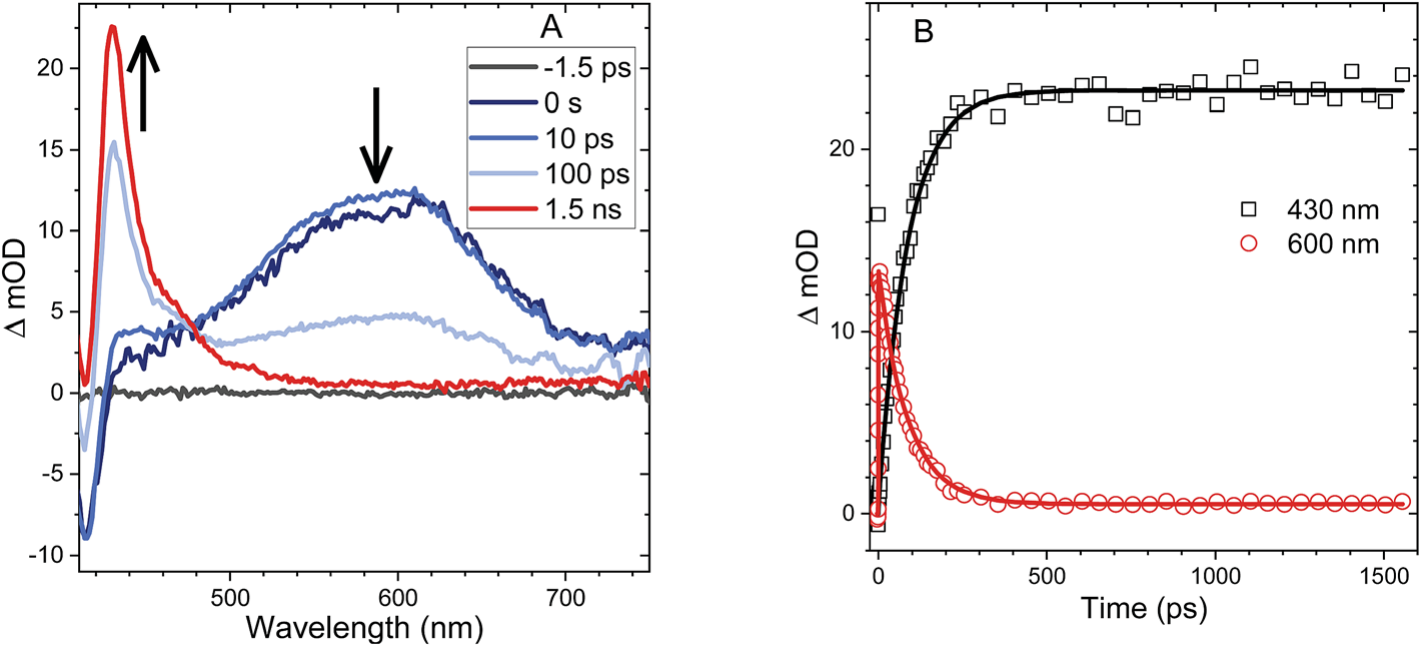
(A) TA spectra of **An–S–An** in acetonitrile showing singlet induced absorption at early times (600 nm) and a ground state bleach (420 nm). At later times, the triplet induced absorption is the dominant feature (430 nm). An isosbestic point is observed between the singlet and triplet features. (B) Kinetic traces corresponding to the singlet induced absorption (600 nm, red circles) and the triplet induced absorption (430 nm, black squares). Both traces are fit with an exponential function (solid lines) having a time constant of 87 ps consistent with the PL lifetime.

Femtosecond transient absorption (fs-TA) can help elucidate the mechanism which leads to rapid PL quenching in all S-bridged molecules. The behavior of all S-bridged compounds is similar. A representative example of the TA data is shown in Fig. 3A for **An–S–An** in acetonitrile. At early times, a broad positive feature centered at ∼600 nm is assigned to the singlet S_1_ → S_N_ induced absorption. By 30 ps a new positive feature emerges at ∼430 nm, similar to the triplet T_1_ → T_N_ feature observed in **An** and its derivatives.^66^–^68^ By 100 ps the T_1_ → T_N_ feature dominates the spectrum while the singlet absorption has decayed. In cyclohexane and CH_2_Cl_2_ the same features emerge in the TA spectra, but the triplet transition appears more slowly, mirroring the trend found in the PL decays (ESI, Fig. S7†). In all solvents, a clear isosbestic point between the singlet and triplet induced absorptions is seen, indicating that a population transfer is occurring between these two well-defined electronic states. A global analysis of the TA data for both **An–S–An** and **An–S–Ph** yields a fit that best describes the data with two species associated spectra (ESI, Fig. S7 and S8†). One spectrum corresponds to the singlet feature which decays with a lifetime closely matching the PL lifetime. The second spectral component has an extremely long lifetime and represents the triplet feature which does not decay during the experiment. An example of this fitting of the fs-TA decays is shown in Fig. 3B. For all the S-bridged compounds, ISC appears to be the dominant mechanism that removes the S_1_ population.

**Fig. 4:**
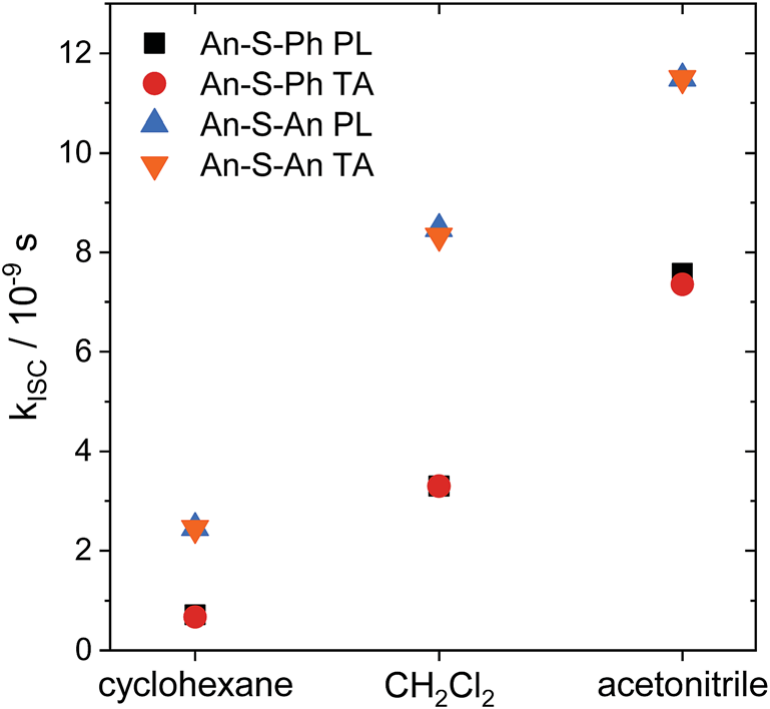
Intersystem crossing rates (*k*_ISC_) as a function of solvent polarity (cyclohexane, *ε* = 2.02; CH_2_Cl_2_, *ε* = 8.93; acetonitrile, *ε* = 36.64)^83^ for **An–S–Ph** calculated from the PL lifetimes (black squares) and TA lifetimes (red circles) compared with the rates for **An–S–An** calculated from the PL lifetimes (blue up triangles) and TA lifetimes (orange down triangles). Intersystem crossing is faster in more polar solvents and faster for **An–S–An** than for **An–S–Ph**.

The relaxation constants measured for **An–S–Ph** and **An–S–An** in the different solvents are summarized in Table 1. For all compounds, the time constants obtained from independently fitting the PL decays and the fs-TA evolution are the same to within 10%. This good correspondence indicates that we are not missing an important relaxation channel in our analysis. In all of the S-bridged compounds, singlet state relaxation is dominated by ISC and we assume that the singlet decay rate reflects the ISC rate (*k*_ISC_). The increase in *k*_ISC_ with increasing solvent polarity for **An–S–An** and **An–S–Ph** is shown in Fig. 4. Qualitatively, this behavior is consistent with the involvement of a singlet state that is stabilized in polar solvents and by the presence of a larger, more polarizable chromophore. These trends, combined with the fluorescence spectra in Fig. 1A, suggest that the singlet state has appreciable CT character. As the energy of this state decreases, it moves closer to the low-lying triplet of **An** and presumably facilitates ISC. Note that if the bridging S atom was merely an innocent bystander that only contributed to electronic screening of the **An–An** interaction (*i.e.* analogous to the terthiophene system) we would expect **An–S–Ph** and **An–S–An** to exhibit behavior similar to monomeric **An**. The large deviations already indicate that the **An** dimer system behaves differently from the terthiophene system.

**Table 1:** Excited singlet state lifetimes of each molecule determined from a single exponential fit of the photoluminescence (PL) and transient absorption (TA) time traces. The S-bridged molecules show good agreement between the different methods. The lifetimes for **An–SO_2_–Ph** and **An–S–Ph** in cyclohexane are too long to be accurately determined from the TA data. The PL lifetimes for **An–SO_2_–An** correspond to the long-lived emission, while the TA lifetimes correspond to the bleach recovery at 400 nm. A reliable bleach recovery lifetime for **An–SO_2_–An** in cyclohexane could not be determined

		Cyclohexane	CH_2_Cl_2_	Acetonitrile
**An–S–Ph**	PL (ps)	1390 ± 5	303 ± 5	132 ± 3
	TA (ps)	1743	280 ± 110	136 ± 97
**An–S–An**	PL (ps)	407 ± 5	118 ± 2	87 ± 2
	TA (ps)	416 ± 153	128 ± 24	84 ± 16
**An–SO_2_–Ph**	PL (ps)	3560 ± 6	229 ± 2	130 ± 2
	TA (ps)	>1500	226 ± 6	144 ± 13
**An–SO_2_–An**	PL (ns)	11.2 ± 0.12	16.8 ± 0.5	10.9 ± 0.03
	TA (ps)	NA	23.8 ± 0.7	19.6 ± 0.3

### 3.3 Excited state dynamics of **An–SO_2_–Ph** and **An–SO_2_–An**

**Fig. 5:**
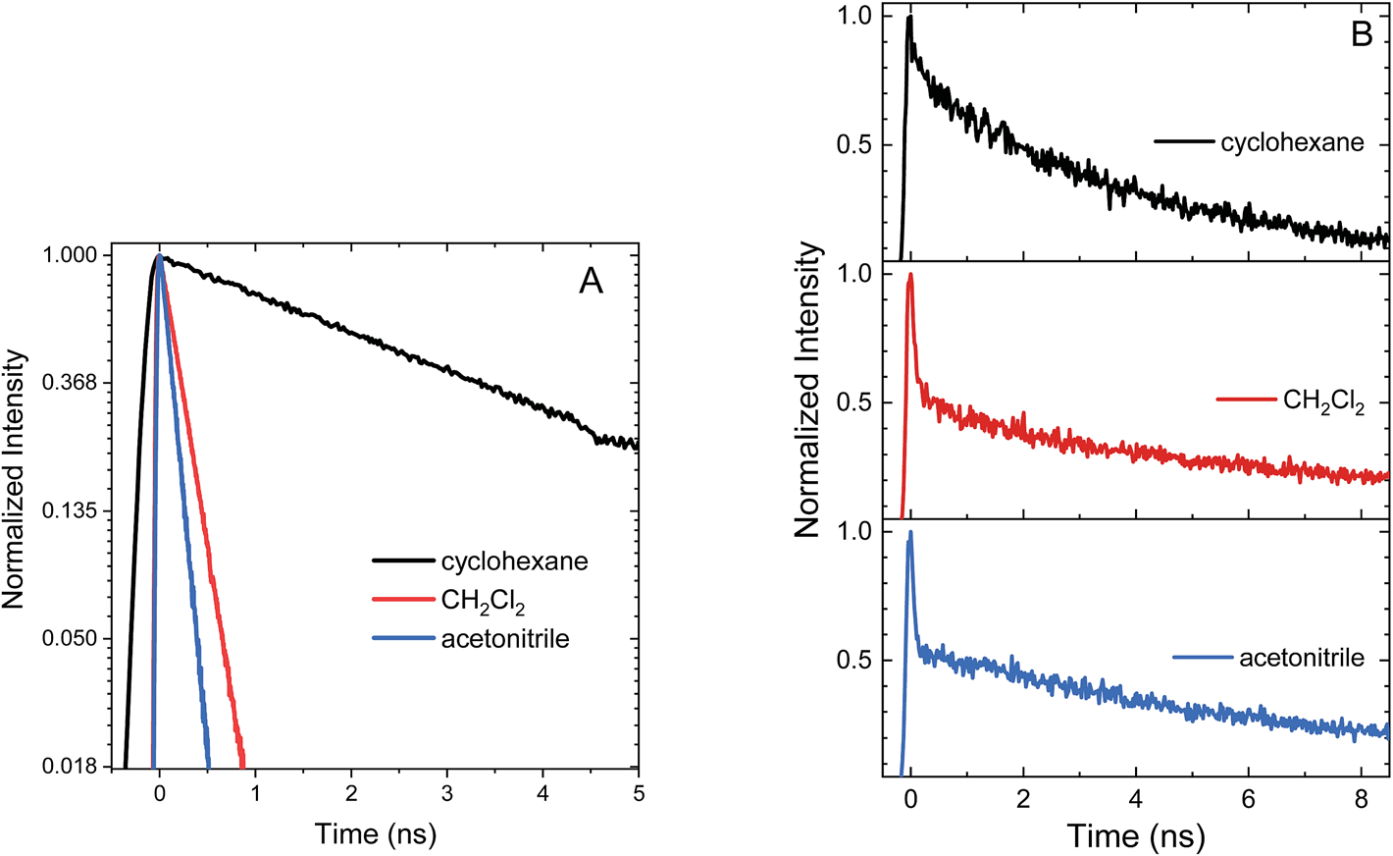
Normalized PL time traces integrated over all wavelengths for (A) **An–SO_2_–Ph** shown on a natural log scale and (B) **An–SO_2_–An** in cyclohexane, CH_2_Cl_2_, and acetonitrile.

The SO_2_-bridged compounds (**An–SO_2_–Ph** and **An–SO_2_–An**) behave similarly to the S-bridged compounds in that their PL decays also depend on solvent polarity. The PL time traces of **An–SO_2_–Ph** are mono-exponential with lifetimes of 3.6 ns in cyclohexane, 230 ps in CH_2_Cl_2_ and 130 ps in acetonitrile (Fig. 5A). However, the PL time traces for **An–SO_2_–An** are biexponential in all solvents, with a prompt decay component that cannot be resolved within the instrument response function of the streak camera. The long-lived tail of the decay has the same emission spectrum as the prompt component but with a lifetime on the order of 10 ns, accounting for roughly 50% of the total decay amplitude (Fig. 5B).

**Fig. 6:**
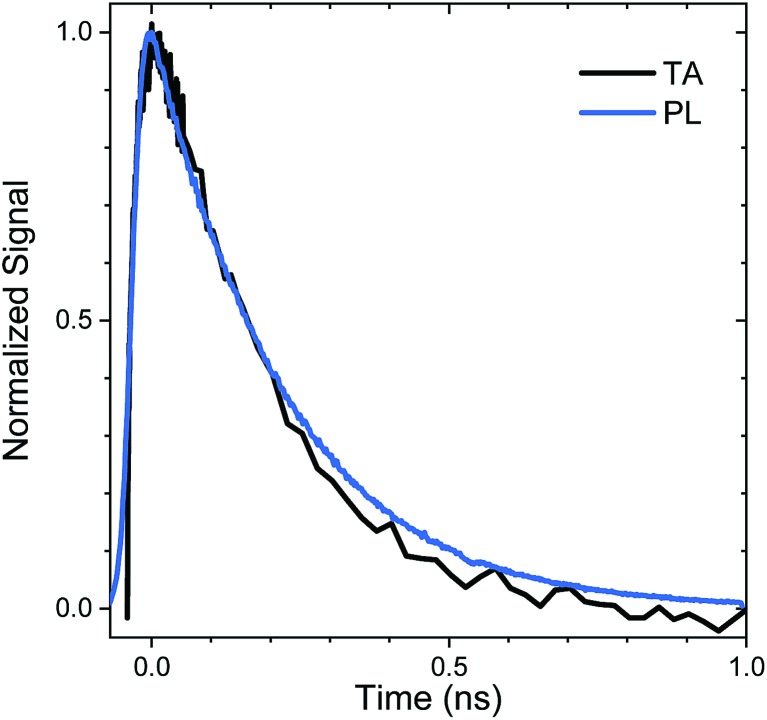
Comparison of the time traces from the transient absorption at 400 nm (TA, black) and photoluminescence (PL, blue) of **An–SO_2_–Ph** in CH_2_Cl_2_. An exponential fit yields a lifetime of 225 ps for both the TA and PL time trace.

The relaxation of the singlet state for the SO_2_-bridged compounds is much faster than that observed for the S-bridged compounds, but ISC is not the culprit in this case. In the TA spectra of **An–SO_2_–Ph**, only a short-lived S_1_ → S_N_ feature is observed in each solvent (ESI, Fig. S9†). In polar solvents there is a slight blue shift of the S_1_ → S_N_ feature, but it disappears on the same time scale as the PL lifetime. No signature T_1_ → T_N_ features appear as the singlet disappears, which indicates that ISC does not play a major role. When probed at 400 nm, the recovery of the ground state absorption mirrors the PL decay, as the comparison in Fig. 6 shows. This correlation suggests that internal conversion to the ground state must be the nonradiative pathway that quenches the PL. The rate of internal conversion becomes more rapid as the solvent polarity is increased. This type of solvent polarity enhanced internal conversion has been previously reported for carotenoid derivatives and attributed to an excited singlet with some CT character.^69^,^70^ It is also possible that the internal conversion proceeds through an intermediate state with CT character whose decay rate is much more rapid than its population rate from the initially excited singlet state (inverted kinetics).^71^–^73^

**Fig. 7:**
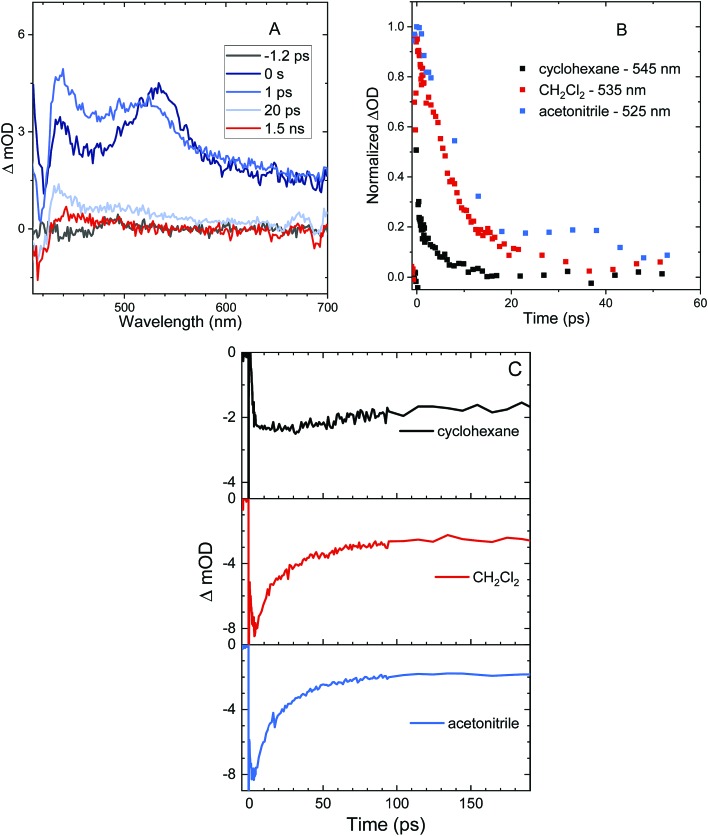
TA spectra of **An–SO_2_–An** in CH_2_Cl_2_ (A) and the time traces showing the early times from the transient absorption spectra of **An–SO_2_–An** in cyclohexane (black), CH_2_Cl_2_ (red) and acetonitrile (blue) at the indicated wavelength (B). The positive features in the TA spectra are due to singlet induced absorption. The negative feature around 420 nm is the ground state bleach which persists throughout the scan. (C) The TA bleach recovery traces at 400 nm in cyclohexane (black), CH_2_Cl_2_ (red), and acetonitrile (blue).

The behavior of the **An–SO_2_–An** bichromophore is more complicated. The femtosecond TA spectra for **An–SO_2_–An** in CH_2_Cl_2_ is shown in Fig. 7A. It is similar to that of **An–SO_2_–Ph**, with broad S_1_ → S_N_ features that are present at early times but disappear in less than 100 ps. Unlike **An–S–An** or **An–S–Ph**, the decay is slower in more polar solvents with the singlet absorption decaying within 3 ps in cyclohexane, 9 ps in CH_2_Cl_2_ and 15 ps in acetonitrile (Fig. 7B). The rapid disappearance of the singlet absorption in the visible spectral range raises the question of where the population may be going. Again, the absence of a T_1_ → T_N_ feature suggests that ISC is not a major factor. To see if the population is returning directly to the ground state as observed in **An–SO_2_–Ph**, we monitored the bleach signal at 400 nm.

Kinetic traces of the 400 nm TA bleach features of **An–SO_2_–An** show that there is a rapid (<100 ps) ground state recovery component in all three solvents, followed by a much longer-lived component. The amplitude of the rapid component is much smaller for cyclohexane than for CH_2_Cl_2_ and acetonitrile (Fig. 7C). The smaller magnitude of the picosecond bleach recovery in cyclohexane is consistent with the PL data in Fig. 5B, where the fast decay component is smaller than in CH_2_Cl_2_ and acetonitrile. The rapid initial decay of the singlet state is limited by the instrument response in the PL measurements, but is clearly resolved in the visible and 400 nm fs-TA data. From the TA and PL data, **An–SO_2_–An** appears to decay by two different pathways. One part of the population relaxes *via* rapid internal conversion directly to the ground state, similar to **An–SO_2_–Ph**. But another portion ends up in an emissive state that survives for 10 ns or longer. Unfortunately, this long-lived state does not appear to have a strong absorption signature in the 400–700 nm range. Based on the broadened emission spectrum, it probably involves CT between the **An** moieties and is the likely precursor for the photocycloaddition reaction between neighboring anthracene rings. Several workers have shown that this type of excimer state can have a strong absorption signature in the near-infrared, and it is possible that a TA experiment that probes this region would reveal the spectroscopic signature of this long-lived state.^74^–^79^

### 3.4 Theoretical analysis

The different behavior of the S- and SO_2_-bridged compounds cannot be rationalized in terms of the screening model previously developed for the terthiophene bichromophores, in which the presence of sulfur lone pairs decreased the amount of excited-state CT character by preventing Coulomb stabilization.^14^ For example, the **An–S–An** excited state appears to have significantly more CT character than **An–SO_2_–An**, which is the opposite of what was observed for the terthiophene compounds. In order to explain these discrepancies, we analyze the excited state structure of the molecules using TD-DFT. Optimization of the molecular ground state geometries is described in the ESI.† All molecules rest in a “displaced” geometry with *C*_2_ symmetry, with the **An** and **Ph** moieties rotated so that the π systems are offset from each other. These structures are consistent with the crystal structure data and there is no evidence that the molecules adopt a significantly different conformation in solution.

#### 3.4.1 Vertical excitations at the Franck–Condon level

**Fig. 8:**
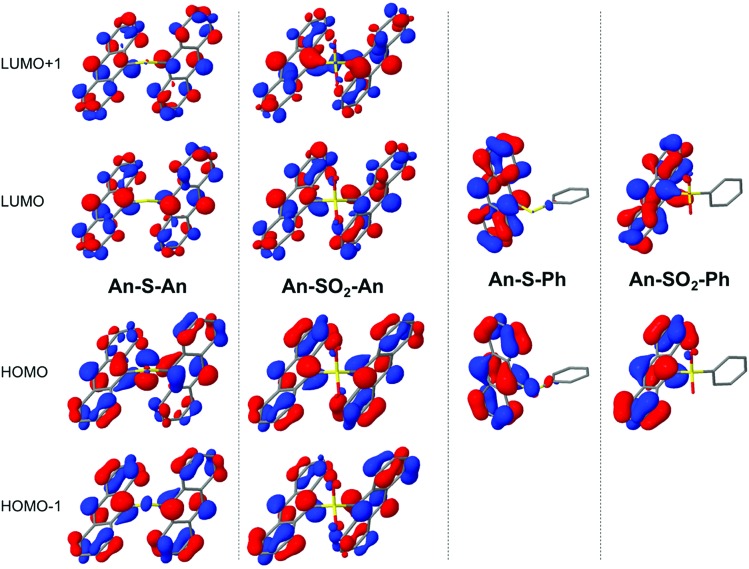
Frontier MOs contributing to the S_0_ → S_1_ electronic transition.

Computed excitation energies to the lowest excited state (Table 2) indicate very similar gaps for the two bridges, in good agreement with the absorption maxima in the steady-state spectra. The nature of the electronic transition can be assessed by means of the frontier molecular orbitals (MOs) (Fig. 8). The highest occupied MO (HOMO) and lowest unoccupied MO (LUMO) of **An–S–An** and **An–SO_2_–An** exhibit electron delocalization over the two anthracene moieties. While frontier MOs of SO_2_-bridged dimers show virtually no involvement of the linker, there is a sizeable contribution of the S lone-pair electrons in the HOMO of **An–S–An**. Moreover, the oxidation state of the linker in **An–S–An** and **An–SO_2_–An** tunes the relative stability of the in-phase and out-of-phase combination of the anthracene frontier MOs, resulting in swapped HOMO/HOMO–1 and LUMO/LUMO+1 character between **An–S–An** and **An–SO_2_–An**. Conversely, the frontier MOs of **An–S–Ph** and **An–SO_2_–Ph** are largely localized on the anthracene fragment. The most dramatic difference between the S- and SO_2_-bridges are the much larger Δ*μ* values for the S-bridged compounds, which implies that these transitions have greater CT character. This is certainly consistent with the pronounced solvatochromism exhibited by **An–S–An** in particular (ESI, Fig. S5†).

**Table 2:** Computed excitation energies (in eV) to the lowest excited singlet state (S_1_), oscillator strength, increase in the dipole moment (in Debyes) upon excitation (Δ*μ* = *μ*(S_1_) – *μ*(S_0_)) and main molecular orbital contributions (in %). H = HOMO, L = LUMO

	Δ*E*	Strength	Δ*μ*	Composition
**An–S–An**	3.46	0.42	–1.01	68 (H → L), 26 (H–1 → L+1)
**An–SO_2_–An**	3.48	0.29	–0.20	53 (H → L), 42 (H–1 → L+1)
**An–S–Ph**	3.65	0.28	–0.70	93 (H → L)
**An–SO_2_–Ph**	3.61	0.25	–0.29	94 (H → L)

**Fig. 9:**
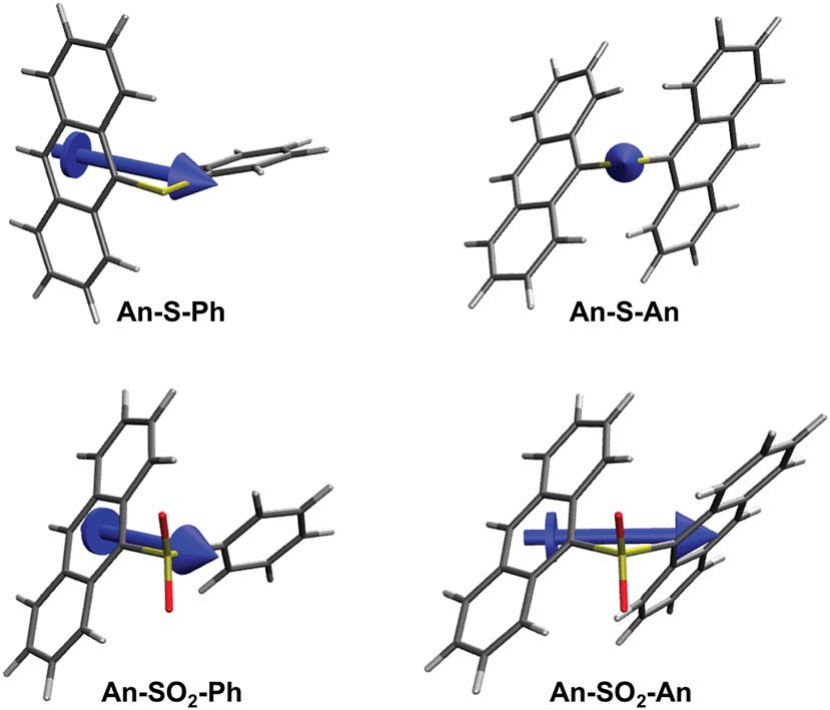
Representation of the transition dipole moments of the S_0_ → S_1_ excitation.

From Table 2, the oscillator strengths for the transition to S_1_ are very similar in all cases, except for **An–S–An**, which has a noticeably larger transition probability. Transition dipole moments (Fig. 9) of both **Ph**-substituted dimers correspond to an **An** localized transition (L_a_ state) with the transition dipole along the **An** short molecular axis. In **An–SO_2_–An**, the orientation of the total transition dipole moment is as expected for an H-type aggregate of two **An** molecules whose transition dipole moments are oriented side-by-side and add out-of-phase. On the other hand, the transition dipole moment to S_1_ in **An–S–An** is oriented perpendicular to that of **An–SO_2_–An** and corresponds to the in-phase combination of local dipoles, as expected for a J-type dimer. Again, this is consistent with the redshift and enhanced 0–0 vibronic peak in the **An–S–An** absorption spectrum (Fig. 1).

Varying the oxidation state dramatically changes the degree of CT and the excitonic coupling between the **An** chromophores (Table 2 and Fig. 9). Enhanced excitonic coupling increases the total oscillator strength of the **An–S–An** transition, which now reflects contributions from both **An** chromophores. To further characterize the nature of the S_1_ state in **An–S–An** and **An–SO_2_–An** and to understand the differences between S and SO_2_ linkers, we decompose the transition to S_1_ in terms of diabatic contributions (ESI†). The lowest state in **An–SO_2_–An** is mainly obtained as the linear combination of local excitations (LE) of the two anthracenes. The excitation to S_1_ in **An–SO_2_–An** is almost purely of LE character, with a very small contribution of π → π* CT between anthracenes. The π → π* CT contributions in **An–S–An** are estimated to account for 5% of the transition, slightly higher than in the SO_2_-bridged dimer. But the S-bridged dimer also receives a sizeable contribution of electron transfer from the lone-pair electrons of the sulfide bridge to the anthracene units, *i.e.* n(S) → π* (CT_B_). The involvement of the lone-pair electrons in the transition to the lowest singlet has been observed in a computational study of a sulfur-bridged naphthalene dimer (**Naph–S–Naph**),^80^ but not for the terthiophene counterpart (**T3–S–T3**).^14^ In **T3–S–T3** the n(S) orbitals lie much lower in energy than the HOMOs of the two terthiophene moieties. As a result, there is no sizeable contribution from n(S) in the transition to lowest excited states of **T3–S–T3**, *i.e.* no relevant n(S) → π* (CT_B_) excitations within the computed lowest 10 excited singlet states.

#### 3.4.2 Excited state relaxation

Computational geometry relaxation on the S_1_ potential energy surface of **An–SO*_x_*–An** dimers results in two structural local minima. The first “displaced” conformation belongs to the *C*_2_ symmetry point group and resembles the ground state minima with modified bond distances according to the π → π* orbital promotions. It is this conformation that is likely reached after the initial relaxation in the S_1_ state. The second set of minima corresponds to the *C*_2v_ symmetry group and involves an “eclipsed” geometry that shows a strong reduction of the C–S–C angle and shorter **An–An** interplane separation, with more coplanar anthracenes forming in the SO_2_-bridged dimer. This geometry is expected to be more conducive to π–π interactions and photodimerization. The detailed structural parameters for all four compounds are given in the ESI.† In both dimers the S_1_ eclipsed form is energetically lower than the displaced disposition, especially for the SO_2_-bridged dimer. Stabilization of the eclipsed conformer is stronger in **An–SO_2_–An** than in the **An–S–An** dimer, indicating stronger electronic coupling between the two **An** units in the former, in agreement with the lone-pair screening interaction model,^14^ that is the weakening of the electronic CT interaction *via* screening by the S lone-pair electrons.

Vertical de-excitation energies from the displaced and eclipsed S_1_ minima are given in the ESI.† The character of the S_1_ emission in the **Ph**-terminated compounds holds strong π → π* character localized on the **An** with a CT_B_ contribution in the S-bridged case (similar to the S_0_ → S_1_ transition at the Franck–Condon region), in agreement with the weak solvent dependence of emission peak maxima. The computed Stokes shifts are on the order of 0.6–0.7 eV, and transition strengths are close to the values computed at the ground state geometry. When the **Ph** group is replaced by **An**, calculations also predict strong Stokes shifts for displaced forms (∼0.9 eV) that are even larger for the eclipsed minima (1.4 eV and 1.8 eV for **An–S–An** and **An–SO_2_–An**, respectively). Furthermore, the eclipsed oscillator strengths are considerably lower with respect to the strengths at the absorbing Franck–Condon region. All the calculated Stokes shift values are much larger than the experimentally observed Stokes shifts in Fig. 1, and it is likely that our TD-DFT calculations overestimate the energy differences between the absorbing and relaxed geometries. The important point is that theory shows that there are two distinct excited state minima for the **An–SO*_x_*–An** compounds, both with diminished oscillator strengths, in contrast to the single relaxed state in the **An–SO*_x_*–Ph** compounds.

#### 3.4.3 Relaxation of S-bridged dimers

Both **An–SO*_x_*–An** dimers can access two conformations, one of which may be a precursor to the photodimer, but the question is whether the initially excited states can survive long enough to populate the eclipsed conformation. For **An–S–An**, the experimental answer is no. Theoretically, the S lone pairs open up several distinct nonradiative relaxation pathways. The excited state singlet of the S-bridged dimers can decay by stabilizing the CT contributions from the S lone-pairs to **An**, as recently discussed for S-bridged naphthalene dimers.^80^ Structural rearrangement in this direction evolves towards an n(S) → σ* state in which the angle between the two **An** moieties becomes linear (C–S–C angle of 180°), the S–C bonds elongate in order to stabilize the σ* orbital, and the gap between ground state and excited state singlets shrinks considerably. The relaxation of the n(S) → σ* state (elongated form) for the S-bridged molecules ultimately results in a small S_1_/S_0_ energy difference that can reach a state crossing (conical intersection), thereby allowing the efficient funneling of the excited system to the ground state. Non-radiative decay through a conical intersection has been suggested as a viable channel in similar S-bonded molecules^80^,^81^ and would only be available in the sulfide dimers, but not in the SO_2_-bridged systems. However, internal conversion to S_0_ does not appear to play a significant role in our S-bridged molecules, which mainly undergo ISC as shown in Section 3.2.

To efficiently relax to the triplet state *via* ISC, two requirements need to be satisfied, that is: (1) a small energy gap between initial (singlet) and final (triplet) states, and (2) sizeable spin–orbit coupling (SOC). At the local excited state minimum the displaced conformer of **An–S–An** presents a large singlet–triplet gap (Table 3). The S_1_–T_1_ relative energy is also large for the **Ph**-substituted and the eclipsed form of **An–S–An**, while the energy difference with respect to T_2_ is reduced, although for **An–S–Ph** the ISC process is energetically uphill. The computed SOCs to T_1_ and T_2_ for the S-bridged dimers are considerably larger than for anthracene and the SO_2_-bridged compounds. Interestingly, the elongated conformer is calculated to have an extremely large SOC (159 and 178 cm^–1^ for **An–S–An** and **An–S–Ph** respectively), suggesting that it could also play a role in ISC. These results, which can be rationalized by means of El-Sayed's rule^82^ (ESI†), support the idea of faster ISC in S-bridged dimers. Moreover, the CT_B_ character of the n(S) → π* and n(S) → σ* orbitals agrees with the solvent polarity dependence of the ISC rate observed in Fig. 4 for the S-bridged compounds.

**Table 3:** Excited singlet-triplet energy gaps (in eV) and SOC (in cm^–1^) between S_1_ at the two lowest triplet states (T_1_ and T_2_) of S- and SO_2_-bridged dimers at the excited state minima and at the crossing point. For the sake of completeness, the values of pristine anthracene have also been included

	S_1_/T_1_		S_1_/T_2_	
	Δ*E*	SOC	Δ*E*	SOC
Anthracene	1.63	0.00	0.02	0.01

**An–S–Ph**				
Displaced	1.48	2.63	–0.23	1.75
Elongated	0.53	0.82	0.27	178.37

**An–S–An**				
Displaced	1.04	2.68	0.89	8.23
Eclipsed	0.98	0.02	0.27	4.10
Elongated	0.52	2.74	0.31	158.59

**An–SO_2_–Ph**				
Displaced	1.48	0.15	–0.17	1.07
Eclipsed	1.46	0.06	–0.28	1.25

**An–SO_2_–An**				
Displaced	1.07	0.30	0.92	1.15
Eclipsed	0.89	0.01	0.02	0.57

#### 3.4.4 Relaxation of SO_2_-bridged dimers

**Fig. 10:**
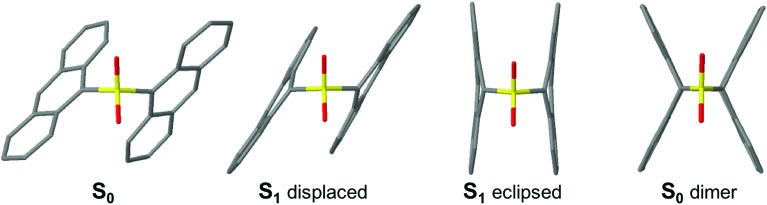
Optimized geometries of **An–SO_2_–An**. From left to right: S_0_, S_1_ displaced, S_1_ eclipsed and S_0_ dimer.

The absence of S lone pairs eliminates possible n(S) → π* and n(S) → σ* contributions to ISC and internal conversion for **An–SO_2_–Ph** and **An–SO_2_–An**. Thus, the formation of a stable, long-lived emissive species in **An–SO_2_–An** is not surprising based on the calculations. The long-lived state probably corresponds to the displaced conformation, judging by the similarity of the emission spectra at early (0–1 ns) and later (1–9 ns) times (ESI, Fig. S11†). On even longer timescales, we speculate that this state interconverts to the eclipsed form. In the eclipsed conformation, the molecular geometry presents relatively short C···C distances (3.2 Å between the S-bonded carbons) and C···C bonding interactions in the excited state (Fig. 10). This conformer is well-positioned to undergo a photocycloaddition reaction between **An** moieties, generating the photodimerized product observed in our previous studies.

While the lack of ISC explains how **An–SO_2_–An** can support a long-lived singlet state, the time-resolved measurements indicate that both SO_2_-bridged compounds experience rapid internal conversion to the ground state as well. The origin of the fast internal conversion channel that dominates the **An–SO_2_–Ph** relaxation and accounts for a substantial fraction of the **An–SO_2_–An** relaxation is intriguing. The ability of the SO_2_ group to induce rapid internal conversion, especially in **An–SO_2_–Ph**, is unexpected since this highly oxidized group should be electronically inert. Searches on the excited state potential energy surfaces of these two molecules uncovered no obvious signs of conical intersections that would provide a path to the ground state. However, the solvent dependence shows that the partitioning of population to the ground state is enhanced in polar environments, suggesting an intermediate with CT character may be involved. One scenario is that the SO_2_ bridge can support different types of CT states in solution, possibly due to relaxation into different conformations. If one CT conformation undergoes rapid charge recombination on a sub-nanosecond timescale, it would lead to an apparent rapid internal conversion, as described above. This conformation would be dominant for **AnSO_2_Ph**, but only about 50% for the **AnSO_2_An** molecule, with the remainder of the population residing in a CT conformation that supports the long-lived emission and the photodimerization reaction. Locating these different excited state conformations would be a considerable challenge for theory, so this explanation must be regarded as tentative for now.

#### 3.4.5 Comparison of S- and SO_2_-bridged relaxation dynamics

**Fig. 11:**
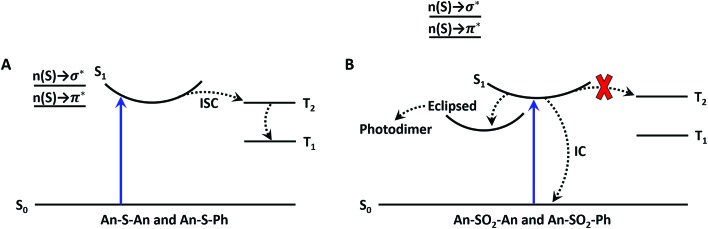
Jablonski diagram for the sulfide (A) and sulfone (B) bridges. The blue upward arrow represents photoexcitation. The dashed lines represent the nonradiative processes of intersystem crossing (ISC) and internal conversion (IC). Mixing of the n(S) → σ* and n(S) → π* linker orbitals enables ISC in the sulfide compounds; whereas, the lack of mixing in the sulfone compounds prevents ISC from occurring.

A simplified schematic of the photophysical behavior of the S- and SO_2_-bridged bichromophores is given in Fig. 11. The key difference between the two bridges is the participation of the sulfur atomic orbitals, which leads to a greater degree of CT in the **An–S–An** excited state and a rapid ISC rate that is sensitive to solvent polarity. The absence of the sulfur orbital contribution in **An–SO_2_–An** allows this molecule to avoid ISC, but it is still subject to a solvent-dependent internal conversion channel of undetermined origin that competes with relaxation to a long-lived emissive state. This long-lived excited state is the likely precursor to formation of the eclipsed isomer which can undergo photodimerization. The key finding is that by tuning the electronic structure of the linker atom, we can dramatically modulate the relaxation pathways of the dimer and enable photochemistry.

## 4. Conclusions

The ability to tune the oxidation state of the sulfur bridge provides a way to investigate the role of the bridge electronic structure in modulating electronic interactions between chromophores. When the bridge and chromophore orbitals are closer in energy, as in the case of anthracene, orbital mixing can lead to unexpected effects, as seen for **An–S–An**, where this mixing leads to enhanced excited state CT character, a change from H-type to J-type excitonic coupling, and rapid ISC. When the bridge orbitals are shifted away by bridge oxidation, behavior is recovered that is more representative of two independent chromophores that interact primarily *via* through-space Coulomb terms. In this case, the bridge affects the excitonic state primarily through geometrical factors and electronic screening, as demonstrated in our previous work on terthiophene dimers.^14^ The present work demonstrates the ability of the linker to tune not only the photophysical but also the photochemical properties of the covalent assembly. In **An–SO_2_–An**, tying up the lone pairs on the S prevents them from short-circuiting the photochemistry by ISC. The ability to engineer the photophysical and photochemical properties of molecular assemblies by tuning the linker electronic structure may prove useful for the design of functional organic optoelectronic materials.

## Conflicts of interest

There are no conflicts of interest to declare.

## Supplementary Material

Supplementary information
