# Supplementary material for: Using sulfur bridge oxidation to control electronic coupling and photochemistry in covalent anthracene dimers
Source: Chem Sci. 2019 Jun 17;10(32):7561–73. doi: 10.1039/c8sc05598j (PMC6713866; doi:10.1039/c8sc05598j)
Supplement: Supplementary file 1 [file SC-010-C8SC05598J-s001.pdf]

Electronic Supplementary Material (ESI) for Chemical Science.
This journal is © The Royal Society of Chemistry 2019

Supplementary Information

Using Sulfur Bridge Oxidation to Control Electronic Coupling and Photochemistry in Covalent Anthracene Dimers

Chad D. Cruz[1], Jennifer Yuan[2], Clàudia Climent[3], Nathan Tierce[1], Peter R. Christensen[2], Eric L. Chronister[1], David Casanova[4]*, Michael O. Wolf[2]*, Christopher J. Bardeen[1]*

[1] Department of Chemistry, University of California Riverside, 501 Big Springs Road, Riverside, California 92521, United States

[2] Department of Chemistry, University of British Columbia, 2036 Main Mall, Vancouver, BC, Canada V6T 1Z1

[3] Departamento de Física Teórica de la Materia Condensada, Universidad Autónoma de Madrid, E-28049 Madrid, Spain

[4] Donostia International Physics Center (DIPC), Paseo Manuel de Lardiazabal 4, 20018 Donostia, Euskadi, Spain; IKERBASQUE, Basque Foundation for Science, 48013 Bilbao, Euskadi, Spain

Synthetic Details

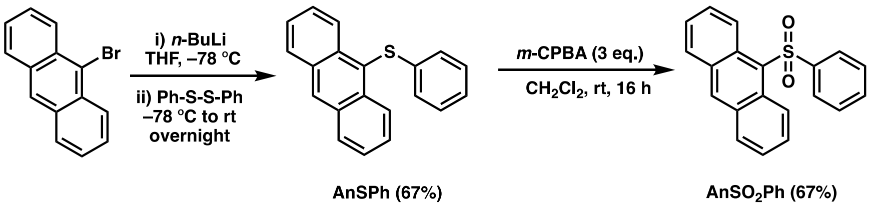

Scheme S1. Reaction conditions for the synthesis of model compounds ($AnSO_nPh$).

9-bromoanthracene was purchased from TCI and meta-chloroperoxybenzoic acid (mCPBA) was purchased from Sigma Aldrich. All other chemicals and solvents were purchased from Sigma Aldrich and used as received. Dry and degassed solvents were obtained from an Innovative Technology solvent purification system solvent purification system. All nuclear magnetic resonance (NMR) spectroscopy was performed on a Bruker 400 MHz spectrometer in $CDCl_3$.

Infrared (IR) spectroscopy was performed on a Perkin-Elmer Frontier FT-IR spectrometer equipped with an attenuated total reflection (ATR) crystal.

Anthracen-9-yl(phenyl)sulfane (AnSPh). In an oven-dried Schlenk flask, 9-bromoanthracene (2.00 g, 7.78 mmol, 1.0 equiv.) was dissolved in dry THF (30 mL) and cooled to –78 °C. To the reaction mixture, *n*-BuLi (1.6 M in hexanes, 5.84 mL, 9.32 mmol, 1.2 equiv.) was added dropwise at –78 °C and allowed to stir for 1 h. In a separate vessel, diphenyl disulfide (1.69 g, 7.78 mmol, 1.0 equiv.) was dissolved in dry THF (10 mL) and added to the reaction in one portion at –78 °C. The reaction was allowed to stir for 30 mins before being warmed to room temperature and left overnight. The reaction was quenched with water (50 mL) and extracted with CH_2Cl_2 (3 × 50 mL). The organic layers were combined, washed with brine, dried over anhydrous $MgSO_4$, filtered and concentrated *in vacuo*. The residue was purified by flash column chromatography on silica gel using hexanes as the eluent to obtain **AnSPh**, a yellow solid. Yield: 1.48 g (67%). ^{1}H NMR (400 MHz, $CDCl_3$): δ 8.88 – 8.81 (m, 2H), 8.64 (s, 1H), 8.13 – 8.05 (m, 2H), 7.61 – 7.51 (m, 4H), 7.15 – 7.08 (m, 2H), 7.07 – 7.01 (m, 1H), 6.98 – 6.92 (m, 2H). ^{13}C{^{1}H} NMR (101 MHz, $CDCl_3$): δ 138.74, 135.02, 131.99, 130.23, 128.93, 128.87, 127.27, 126.86, 126.23, 125.59, 125.23, 124.88. HR-EI-MS: *m/z* calcd for $C_{20}H_{14}S$ 286.08162, found 286.08175.

9-(Phenylsulfonyl)anthracene ($AnSO_2Ph$). In a RBF, (0.300 g, 1.05 mmol, 1.0 equiv.) was dissolved in CH2CL2 (15 mL) and *m*-CPBA (0.555 g, 3.17 mmol, 3.0 equiv.) was added in one portion. The reaction was allowed to stir at room temperature overnight and was monitored using TLC (approximately 16 h). The reaction was quenched with a solution of saturated $NaHCO_3$ (aq) (30 mL) and extracted with CH_2Cl_2 (3 × 20 mL). The organic layers were combined, washed with

brine, dried over anhydrous $MgSO_4$, filtered and concentrated *in vacuo*. The residue was purified by flash column chromatography on silica gel using CH_2Cl_2:hexanes (1:1) as the eluent to obtain **$AnSO_2Ph$**, a yellow solid. Yield: 0.213 g (67%). ^1H NMR (400 MHz, $CDCl_3$): δ 9.44 (m, 2H), 8.78 (s, 1H), 8.11 – 8.05 (m, 2H), 8.01 – 7.96 (m, 2H), 7.67 (m, 2H), 7.59 – 7.49 (m, 3H), 7.48 – 7.42 (m, 2H). $^{13}C\{^1H\}$ NMR (101 MHz, $CDCl_3$): δ 144.25, 140.24, 139.08, 136.77, 132.73, 132.64, 131.29, 131.18, 129.46, 129.04, 128.95, 128.91, 128.83, 127.90, 127.22, 126.50, 126.08, 125.90, 125.46, 125.00. HR-EI-MS: *m/z* calcd 318.07145, found 318.07155. IR (neat): $\tilde{\nu}$ ($\sigma(SO_2)$) 1310, 1147 cm^{-1}.

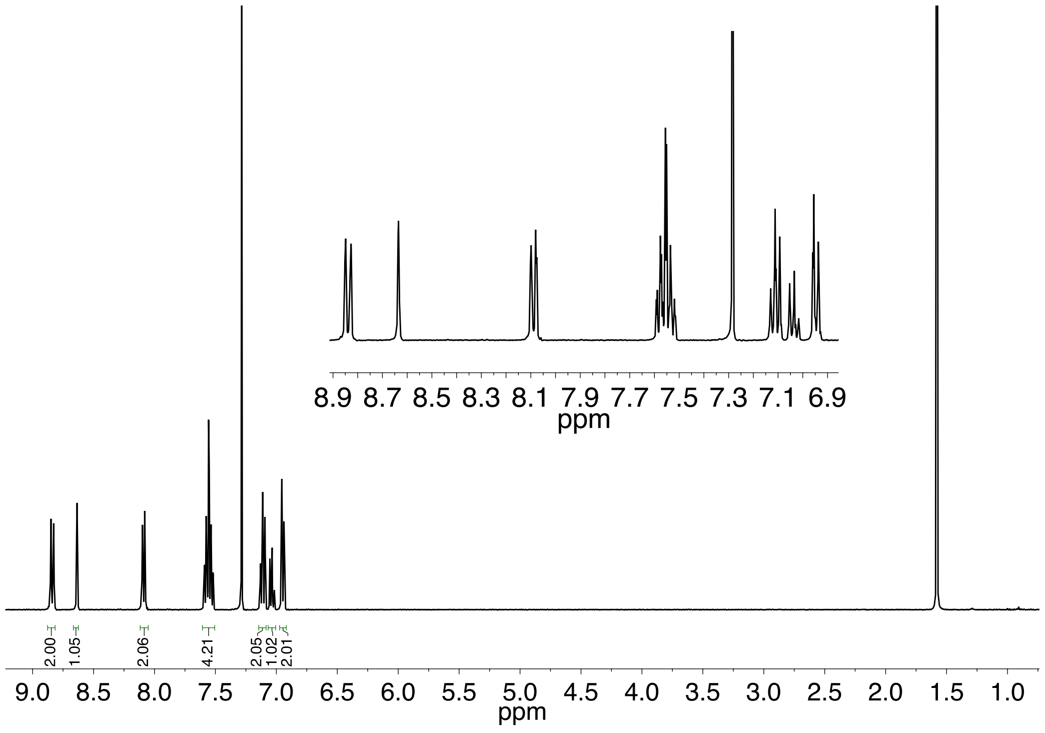

Figure S1. ^1H NMR spectrum of **AnSPh** (400 MHz, $CDCl_3$).

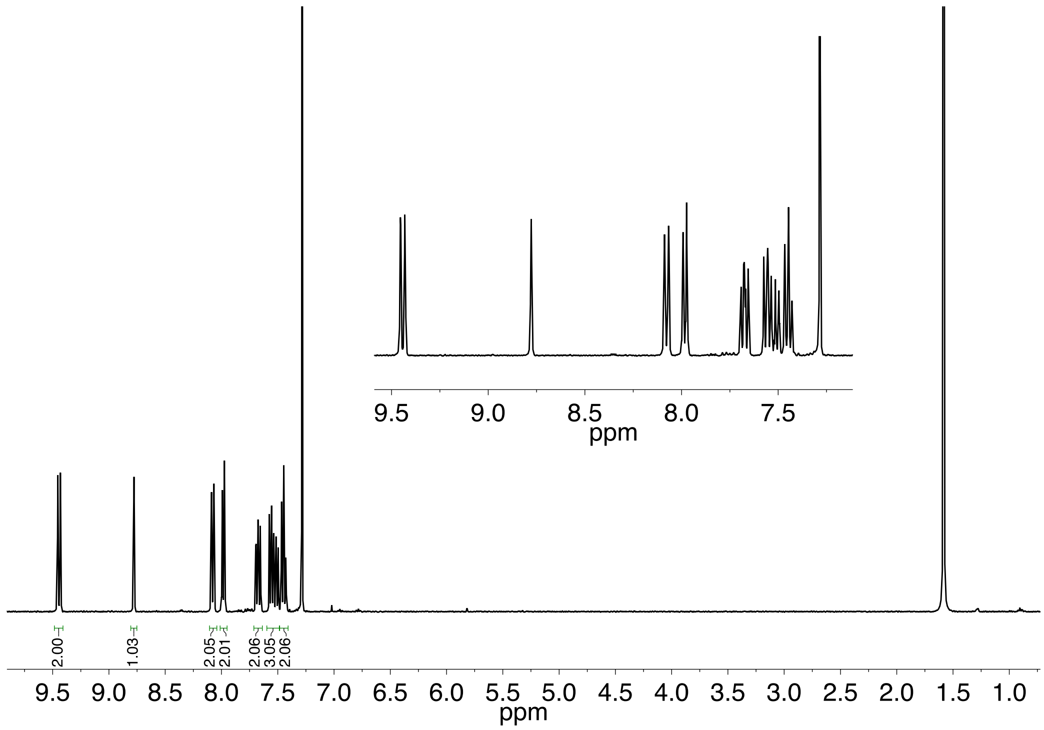

Figure S2. ^{1}H NMR spectrum of **AnSO2Ph** (400 MHz, $CDCl_3$).

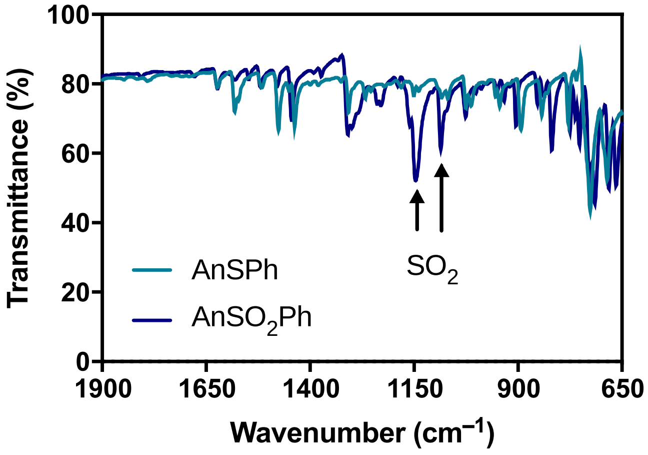

Figure S3. IR spectrum of **An-S-Ph** and **An-SO2-Ph**.

Spectroscopic Details

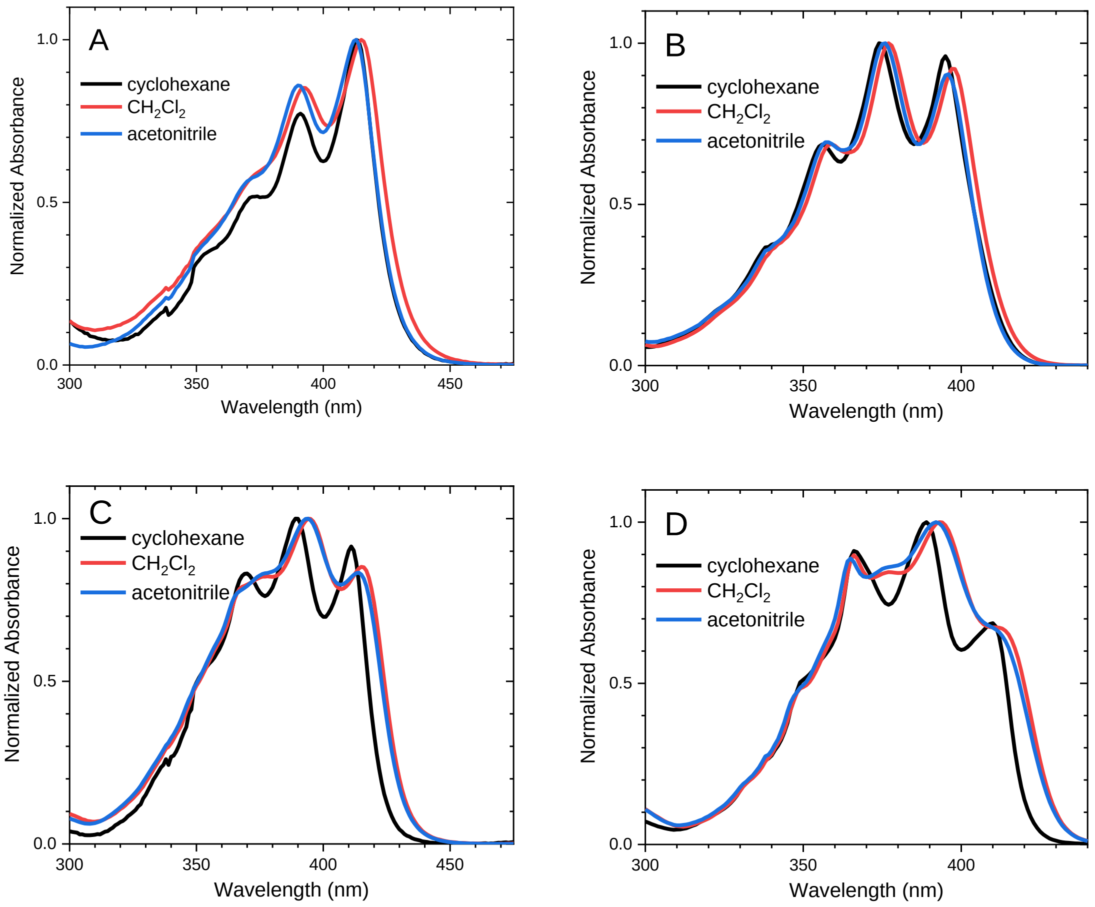

Figure S4. Normalized steady-state absorption of **An-S-An** (A), **An-S-Ph** (B), **An-SO$_2$-An** (C), and **An-SO$_2$-Ph** (D) in dilute solutions of cyclohexane (black), CH_2Cl_2 (red), and acetonitrile (blue).

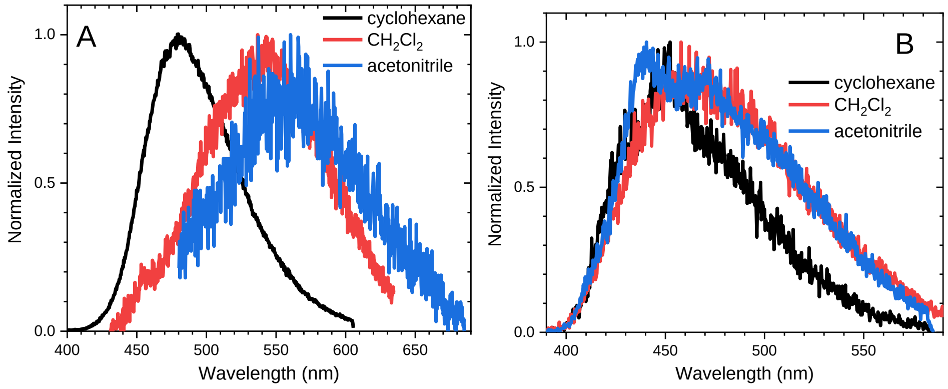

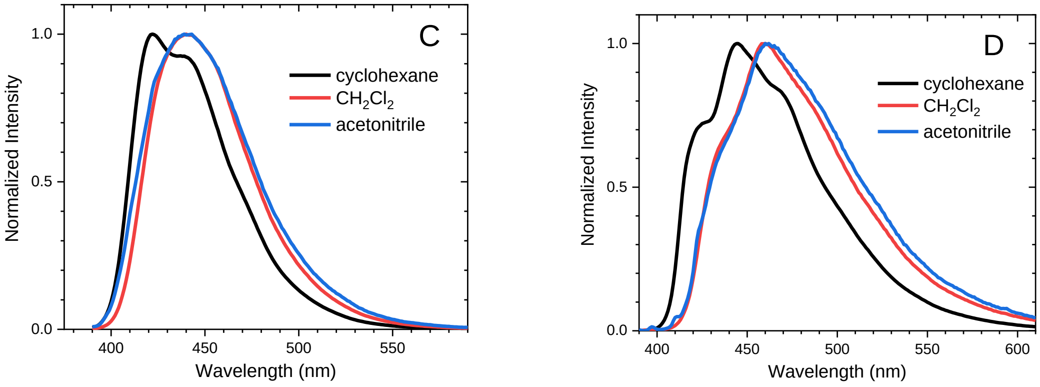

Figure S5. The normalized PL spectra of **An-S-An** (A) and **An-SO$_2$-An** (B) in dilute solutions of cyclohexane (black), CH_2Cl_2 (red), and acetonitrile (blue) showing the PL corresponding to the time traces shown in figures 2B and 5B, respectively. The steady-state PL spectra of **An-S-Ph** (C) and **An-SO$_2$-Ph** (D).

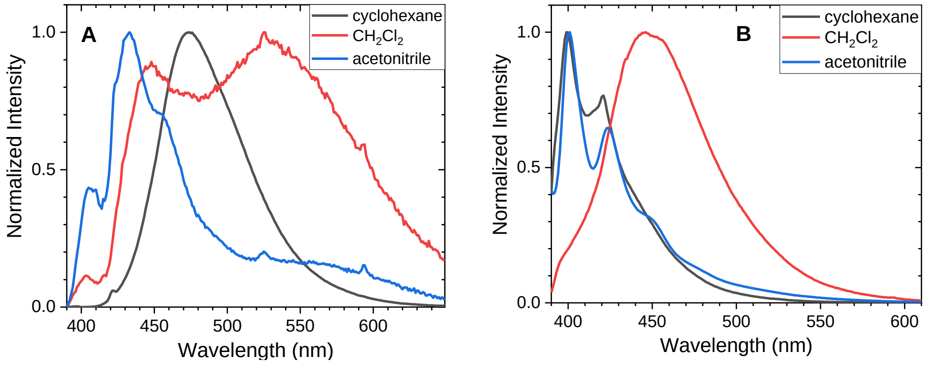

Figure S6. Steady-state emission spectra of An-S-An (A) and An-SO$_2$-An (B) excited at 375 nm. Due to the inherent sensitivity of emission spectroscopy residual impurities can be identified in the emission spectra which are absent in the absorption spectra. The features between 390 – 420 nm overlap the absorption spectra of the dimers (Figure 1) indicating that the origin of these features is an impurity. The most likely culprit is an anthracene derivative used during the synthesis of the dimers. Using time-resolved emission spectroscopy, the impurity emission can be windowed out of the analysis.

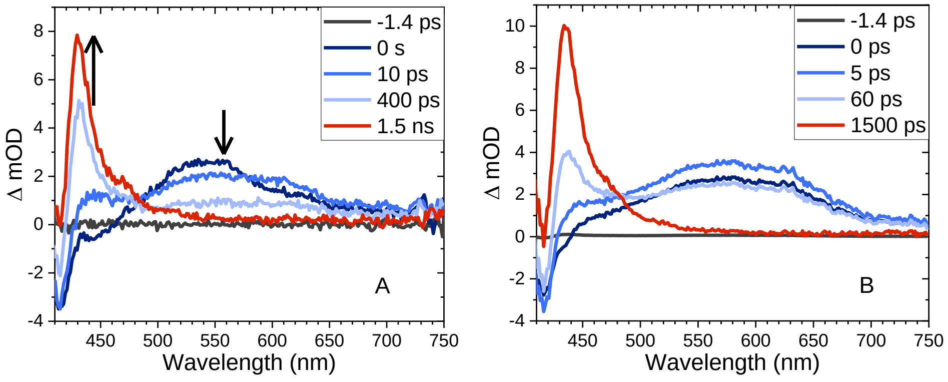

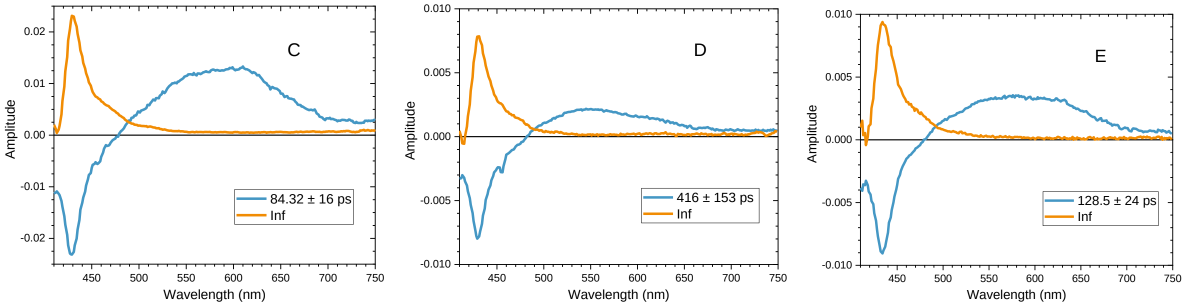

Figure S7. The transient absorption spectra of **An-S-An** in cyclohexane (A) and CH_2Cl_2 (B). The corresponding species associated spectra obtained from a global analysis of the transient absorption data in acetonitrile (C), cyclohexane (D) and CH_2Cl_2 (E).

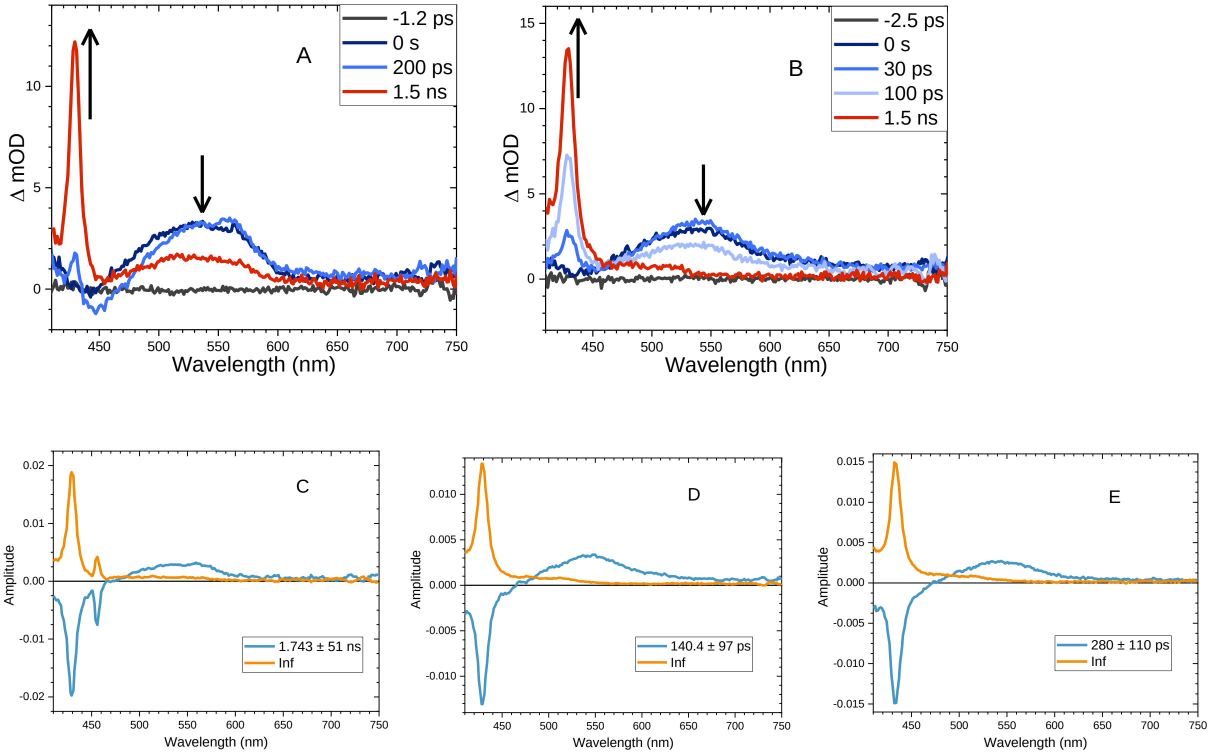

Figure S8. The transient absorption spectra of **An-S-Ph** in cyclohexane (A) and acetonitrile (B). The corresponding species associated spectra obtained from a global analysis of the transient absorption data in cyclohexane (C), acetonitrile (D) and CH_2Cl_2 (E).

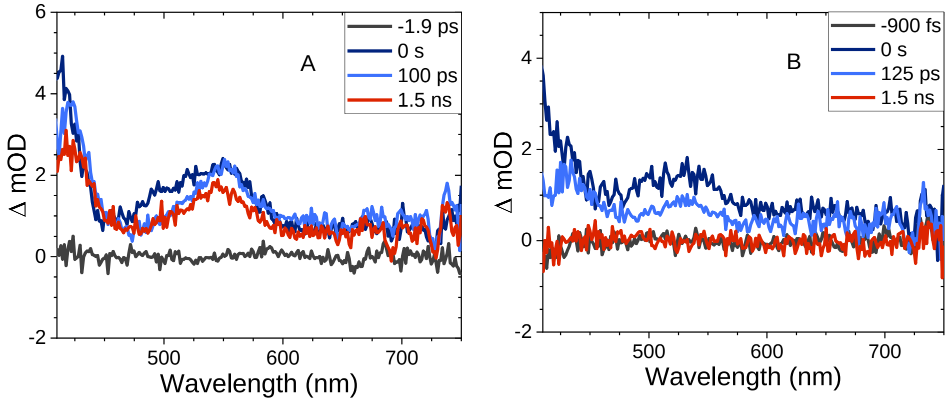

Figure S9. The transient absorption spectrum of **An-SO2-Ph** in cyclohexane (A) and CH_2Cl_2 (B).

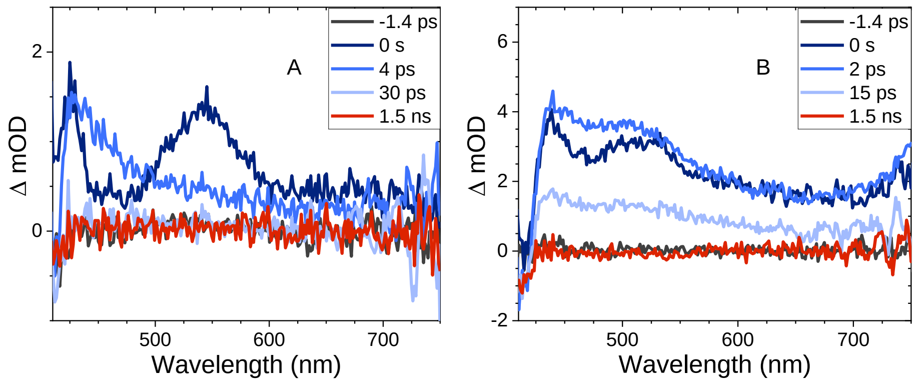

Figure S10. The transient absorption spectrum of **An-SO2-An** in cyclohexane (A) and acetonitrile (B).

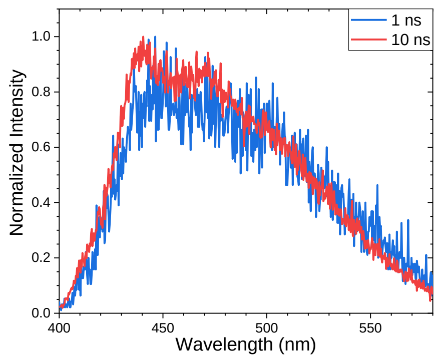

Figure S11. Time-resolved PL spectrum of **An-SO$_2$-An** in acetonitrile showing the signal integrated over 1 ns and 10 ns.

Computational Details

Ground State Molecular structures

Ground state optimized geometries of S and SO_2 bridged anthracene dimers are very similar to each other, exhibiting C_2 symmetry with equivalent anthracene moieties in oblique disposition, C-S-C angle of ~104°, and a dihedral angle between anthracene molecular planes of ~45° (Figure S12). Dimers with a phenyl unit exhibit molecular geometries with larger dihedral angles between chromophoric planes, especially in **An-S-Ph** where the phenyl is oriented almost perpendicularly with respect to the **An** fragment. It is worth noting that SO_2-bridged molecules present relatively short O···H distances, i.e. in the order of 2.1 Å, between the linker and anthracene protons fixing the relative orientation of the two chromophores.

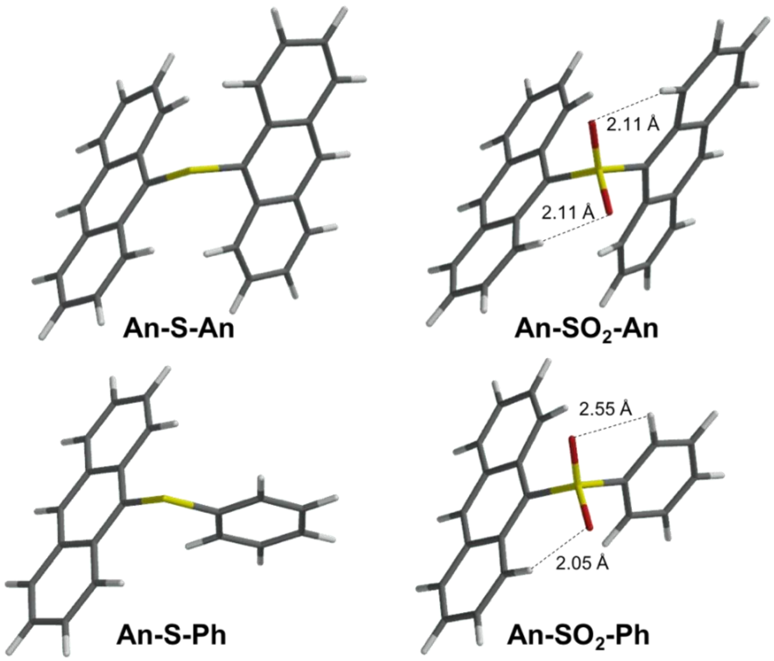

Figure S12. Ground state optimized geometries of **An-S-An**, **An-S-Ph**, **An-SO_2-An** and **An-SO_2-Ph** molecules.

Excited States of Dimers

Table S1. Decomposition (in %) of S_1 of **An-S-An** and **An-SO$_2$-An** dimers in terms of anthracene locally excited (LE), charge transfer between anthracene moieties (CT) and electron transfer from the bridge to the chromophores (CT_B), and electronic couplings (in meV) between LE and CT and CT_B diabatic contributions.

	contributions / %			couplings / meV	
	LE	CT	CT_B	LE/CT	LE/CT_B
An-S-An	76	5	19	51	372
An-SO$_2$-An	99	1	-	40	-

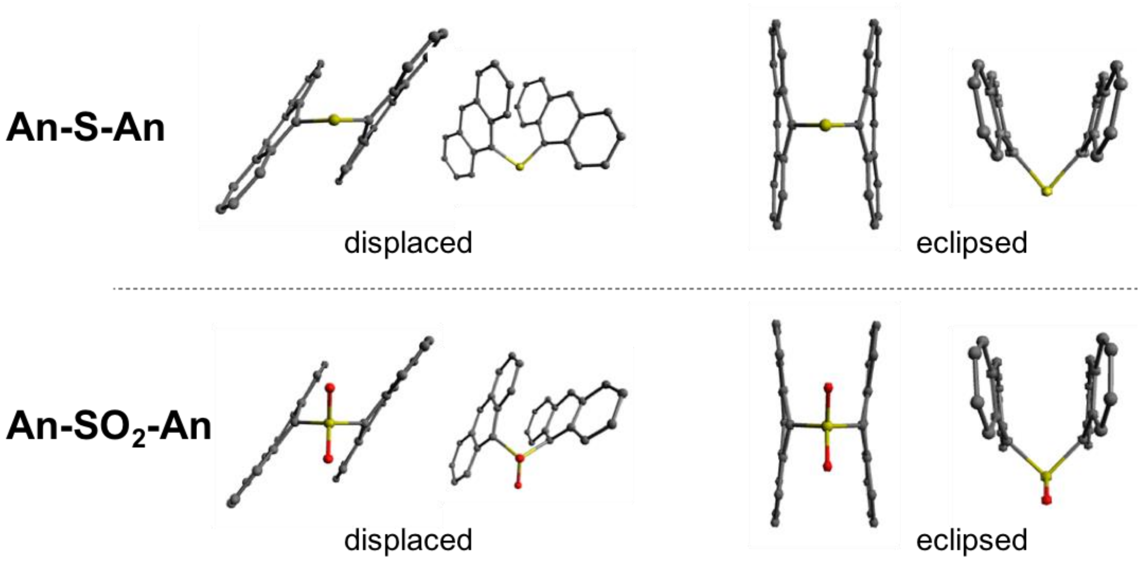

Figure S13. Excited state optimized geometries of displaced (left) and eclipsed (right) for **An-S-An**, (top) **An-SO$_2$-An** (bottom) dimers.

*Structural comparison of **An-SO$_2$-An** displaced and eclipsed forms to FC geometry*

Structural analysis (Table S2) clearly identifies the S_1 displaced form as being structurally much closer to the ground state geometry than the S_1 eclipsed minimum (Figure S13, left), while the S_1 eclipsed minimum is rather close to the dimerization product (Figure S13, right).

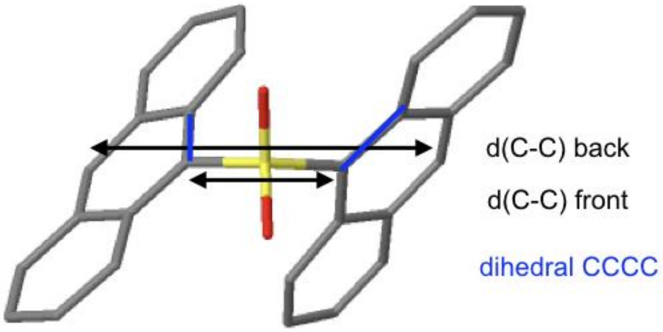

Figure S14. Structural parameters of **An-SO$_2$-An** given in Table S2.

Table S2. Geometrical parameters (Figure S14) for ground state (S_0), S_1 displaced, S_1 eclipsed and S_0 dimer forms of **An-SO$_2$-An**. Distances given in Å and angles in degrees.

parameter	S_0	S_1 displaced	S_1 eclipsed	S_0 dimer
d(C-C) front	2.844	2.794	2.567	1.675
d(C-C) back	6.645	5.226	3.289	1.609
d(C-S)	1.812	1.790	1.774	1.764
angle C-S-C	103.4	102.6	92.6	56.7
dihedral CCCC	53.1	54.7	0.0	0.0

Table S3. Computed deexcitation energies (in eV) from the displaced and eclipsed lowest excited singlet state ($S_1 \rightarrow S_0$) and oscillator strengths computed in DCM solution.

molecule	conformer	character	ΔE	strength
An-S-An	displaced	$\pi\rightarrow\pi^*$	2.57	0.09
	eclipsed	$\pi\rightarrow\pi^*$	2.07	0.12
	elongated	$n(S)\rightarrow\sigma^*$	0.74	0.00
An-SO$_2$-An	displaced	$\pi\rightarrow\pi^*$	2.59	0.01
	eclipsed	$\pi\rightarrow\pi^*$	1.67	0.05
An-S-Ph	displaced	$\pi\rightarrow\pi^*$	3.07	0.32
An-SO$_2$-Ph	displaced	$\pi\rightarrow\pi^*$	3.02	0.25
	eclipsed	$\pi\rightarrow\pi^*$	2.88	0.22

The electronic structure of excimers in interacting chromophores is characterized by strong mixings of exciton and charge resonances, i.e. LE and CT.[1] Decomposition of the electronic transition to S_1 in terms of diabatic state contributions (Table S4) confirms that the displaced and eclipsed relaxed forms can be indeed labeled as excimers. Eclipsed and displaced excimers are

built as even LE and CT contributions with no involvement of the linker, except for the displaced form of **An-S-An**, where the lone-pair electrons of the sulfide bridge play an important role.

Table S4. Decomposition (in %) of S_1 of **An-S-An** and **An-SO$_2$-An** dimers in terms of anthracene locally excited (LE), charge transfer between anthracene moieties (CT) and electron transfer from the bridge to the chromophores (CT_B), and electronic couplings (in meV) between LE and CT and CT_B diabatic contributions.

	contributions / %			couplings / meV
	LE	CT	CT_B	LE/CT
An-S-An				
displaced	37	27	36	199
eclipsed	50	50	0	685
An-SO$_2$-An				
displaced	46	54	-	282
eclipsed	46	54	-	808

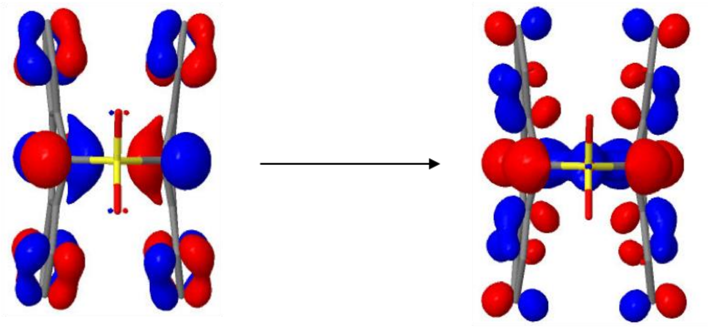

Figure S15. Frontier molecular orbitals of eclipsed excimer of **An-SO$_2$-An** with bonding interactions between **An** in the LUMO.

Spin-Orbit Coupling (SOC) and the El-Sayed's rule

The relative magnitude of the SOC matrix element between S_1 and T_1 or T_2 at the elongated forms (i.e. n(S)→σ*) of the **An-S-An** and **An-S-Ph** dimers can be rationalized by El-Sayed's rule. The S_1 and T_1 states have $n_{\perp}$(S)→σ* character, where the n(S) orbital involved is perpendicular to the anthracene/phenyl plane, i.e. the one which can interact with the π-system. On the other hand, the T_2 state has $n_{//}$(S)→σ* character. Therefore, the largest SOC matrix elements are those between states where the n(S) orbital involved has different symmetry, i.e. S_1 with T_2.

Optimized Geometries

Ground state optimized coordinates for the An-S-An and An-SO_2-An dimers (in Angstroms).

An-S-An coordinates

C	-18.343804	0.652497	-7.639964
C	-21.161848	0.512671	-7.470502
C	-18.149965	-0.589900	-8.282366
C	-21.440678	1.213099	-8.664481
C	-18.953503	-1.749572	-8.028598
C	-20.648344	2.305117	-9.148897
C	-18.747630	-2.919414	-8.703354
C	-20.941453	2.928106	-10.328861
C	-17.721088	-3.027711	-9.686253
C	-22.050465	2.512161	-11.121743
C	-16.909144	-1.960440	-9.935322
C	-22.852306	1.499703	-10.683253
C	-17.084081	-0.721103	-9.237965
C	-22.586581	0.831408	-9.444118
C	-16.220029	0.350284	-9.463853
C	-23.438308	-0.170958	-8.980086
C	-16.363268	1.555988	-8.778490
C	-23.206336	-0.816972	-7.766553
C	-15.450410	2.637560	-9.002505
C	-24.105442	-1.826761	-7.292085
C	-15.597787	3.828114	-8.353953
C	-23.872773	-2.480245	-6.118016
C	-16.679267	4.006947	-7.442390
C	-22.714966	-2.161620	-5.349553
C	-17.569499	2.999210	-7.201380
C	-21.835045	-1.204112	-5.767927
C	-17.449186	1.725447	-7.851739
C	-22.042720	-0.482664	-6.991171
H	-19.725467	-1.706680	-7.271342
H	-19.813090	2.659365	-8.558844
H	-19.368267	-3.782969	-8.481094
H	-20.326591	3.758900	-10.663611
H	-17.580665	-3.964083	-10.218330
H	-22.259905	3.014785	-12.061500
H	-16.105624	-2.029966	-10.664497
H	-23.716233	1.182711	-11.262324
H	-15.411660	0.239610	-10.183669
H	-24.306990	-0.448339	-9.573610
H	-14.635346	2.483349	-9.705406
H	-24.979856	-2.059118	-7.895057
H	-14.900745	4.642355	-8.529325
H	-24.559841	-3.244090	-5.765754
H	-16.796839	4.959966	-6.934229
H	-22.530125	-2.690642	-4.418817

H	-18.384777	3.163039	-6.505246
H	-20.960531	-0.983062	-5.165825
S	-19.704571	0.896438	-6.493869

An-SO_2-An coordinates

C	-41.050426	13.298058	4.810338
C	-41.442549	14.186017	3.779419
C	-41.047184	15.562489	3.686061
C	-41.414546	16.335899	2.620806
C	-42.214188	15.813036	1.565896
C	-42.657008	14.525900	1.638762
C	-42.302452	13.687321	2.743462
C	-42.787830	12.383629	2.819871
C	-42.503018	11.555238	3.902810
C	-43.083481	10.248167	3.971334
C	-42.829956	9.422302	5.025046
C	-41.982876	9.874804	6.075194
C	-41.397388	11.109811	6.042156
C	-41.617765	12.008653	4.943953
C	-38.426149	14.390086	4.726555
C	-38.091136	13.483485	3.691718
C	-38.489800	12.105597	3.645253
C	-38.181582	11.313075	2.575301
C	-37.442121	11.816570	1.468366
C	-36.997285	13.104843	1.493178
C	-37.291061	13.963414	2.600384
C	-36.804479	15.268672	2.625433
C	-37.030346	16.117089	3.706704
C	-36.450229	17.426131	3.717689
C	-36.647168	18.271894	4.767669
C	-37.433494	17.838399	5.871676
C	-38.016521	16.602047	5.895129
C	-37.855063	15.682368	4.803938
H	-40.488848	16.026578	4.487274
H	-41.105640	17.376919	2.591157
H	-42.485566	16.446914	0.727241
H	-43.296291	14.109187	0.864746
H	-43.424799	12.009145	2.021393
H	-43.740225	9.938116	3.162576
H	-43.276455	8.433888	5.076214
H	-41.803444	9.228296	6.929629
H	-40.772752	11.415855	6.867839
H	-39.001975	11.655838	4.484542
H	-38.490869	10.271758	2.581662
H	-37.217000	11.167510	0.627651
H	-36.402440	13.507513	0.677427
H	-36.213829	15.628512	1.785702
H	-35.840176	17.721422	2.867857

H	-36.201247	19.261845	4.774889
H	-37.567049	18.501095	6.722118
H	-38.593265	16.311056	6.760123
O	-39.136505	12.736309	6.655308
O	-40.232062	14.982894	6.668593
S	-39.706306	13.853454	5.891128

Excited state (S_1) optimized coordinates for the An-S-An and An-SO_2-An dimers (in Angstroms).

An-S-An *displaced* coordinates

C	-18.369787	0.667752	-7.331298
C	-21.094235	0.653353	-7.187570
C	-18.505837	-0.411067	-8.275382
C	-21.077091	1.061500	-8.568463
C	-19.225940	-1.592213	-7.993365
C	-20.365164	2.191112	-9.026802
C	-19.466754	-2.541247	-8.969430
C	-20.242026	2.468411	-10.375621
C	-18.953489	-2.366865	-10.273377
C	-20.871494	1.642103	-11.332237
C	-18.183537	-1.265259	-10.563258
C	-21.636625	0.577499	-10.918123
C	-17.914206	-0.272678	-9.574117
C	-21.785577	0.271029	-9.532388
C	-17.094829	0.826384	-9.847464
C	-22.599212	-0.780529	-9.100944
C	-16.775347	1.778439	-8.854843
C	-22.797796	-1.050067	-7.729160
C	-15.864889	2.835666	-9.121496
C	-23.702739	-2.059728	-7.305627
C	-15.583067	3.790456	-8.169529
C	-23.867733	-2.353914	-5.970104
C	-16.216402	3.732677	-6.910837
C	-23.118355	-1.655081	-5.001151
C	-17.118902	2.725308	-6.628947
C	-22.218622	-0.679285	-5.384670
C	-17.415101	1.715466	-7.574803
C	-22.040197	-0.335688	-6.745616
H	-19.620494	-1.749470	-6.993910
H	-19.882404	2.844663	-8.306299
H	-20.039331	-3.431163	-8.725041
H	-19.672139	3.335323	-10.696870
H	-19.153747	-3.110663	-11.039280
H	-20.763520	1.858490	-12.391228
H	-17.756219	-1.135546	-11.554799
H	-22.152329	-0.047323	-11.643536
H	-16.652832	0.928055	-10.836669

H	-23.130928	-1.379235	-9.837855
H	-15.396767	2.881079	-10.102048
H	-24.261492	-2.605187	-8.062548
H	-14.881749	4.590534	-8.388432
H	-24.566485	-3.126639	-5.662665
H	-15.998130	4.485863	-6.159243
H	-23.244763	-1.886818	-3.947513
H	-17.604434	2.707458	-5.656959
H	-21.642833	-0.161831	-4.622066
S	-19.677508	0.967285	-6.194140

An-SO_2-An *displaced* coordinates

C	-18.330450	0.651634	-7.236681
C	-21.120527	0.717332	-7.112975
C	-18.535096	-0.456439	-8.138496
C	-21.033569	1.170402	-8.480464
C	-19.214670	-1.638712	-7.785484
C	-20.351580	2.337250	-8.877611
C	-19.568309	-2.578366	-8.744805
C	-20.115371	2.610723	-10.218447
C	-19.206284	-2.407553	-10.093597
C	-20.603797	1.760843	-11.227877
C	-18.486190	-1.295664	-10.463638
C	-21.330491	0.646687	-10.878831
C	-18.118485	-0.305312	-9.506851
C	-21.578780	0.328660	-9.511677
C	-17.396432	0.823360	-9.895166
C	-22.307386	-0.810276	-9.169275
C	-16.981640	1.793698	-8.957221
C	-22.601856	-1.122164	-7.824230
C	-16.137366	2.859805	-9.345265
C	-23.452601	-2.205364	-7.503313
C	-15.728986	3.809697	-8.430725
C	-23.744020	-2.512887	-6.189687
C	-16.146149	3.715205	-7.089239
C	-23.198731	-1.734429	-5.150770
C	-16.985097	2.694569	-6.685170
C	-22.350715	-0.681119	-5.433413
C	-17.450196	1.716043	-7.603795
C	-22.004029	-0.352912	-6.771457
H	-19.498650	-1.805344	-6.751606
H	-19.971256	3.018897	-8.123923
H	-20.116767	-3.464700	-8.439798
H	-19.560748	3.505345	-10.485752
H	-19.492052	-3.149938	-10.832400
H	-20.409049	1.987096	-12.271695
H	-18.182501	-1.150656	-11.497213
H	-21.730626	-0.015791	-11.642094

H	-17.096723	0.932272	-10.934959
H	-22.705224	-1.445374	-9.957488
H	-15.812304	2.916959	-10.381112
H	-23.876900	-2.792812	-8.313719
H	-15.075629	4.619762	-8.741252
H	-24.403780	-3.343831	-5.957878
H	-15.797732	4.442263	-6.361826
H	-23.454409	-1.954387	-4.118597
H	-17.271311	2.623605	-5.644021
H	-21.963860	-0.078371	-4.622243
S	-19.670211	0.994815	-6.100404
O	-19.609922	2.414610	-5.711801
O	-19.655148	0.001268	-5.012642

An-S-An *eclipsed* coordinates

C	-27.008767	-22.083423	3.768696
C	-27.107620	-20.782915	3.171111
C	-26.986963	-19.584612	3.901371
C	-26.993890	-18.350371	3.267983
C	-27.096097	-18.273273	1.868122
C	-27.230277	-19.427636	1.122273
C	-27.269278	-20.702684	1.746766
C	-27.445311	-21.880817	1.000747
C	-27.619026	-23.128400	1.624384
C	-27.932416	-24.294549	0.876618
C	-28.139570	-25.507714	1.502870
C	-28.038927	-25.601213	2.901870
C	-27.693180	-24.487825	3.653780
C	-27.461373	-23.237923	3.047219
C	-24.704955	-22.428548	4.056091
C	-24.446644	-23.689287	3.422001
C	-24.738893	-24.930528	4.020146
C	-24.561265	-26.122031	3.332211
C	-24.106532	-26.110986	2.002106
C	-23.796936	-24.912714	1.389591
C	-23.928180	-23.680285	2.083176
C	-23.578353	-22.458614	1.482545
C	-23.579037	-21.254575	2.207697
C	-23.096536	-20.045655	1.639441
C	-23.065182	-18.876516	2.373607
C	-23.517577	-18.871507	3.704574
C	-24.033184	-20.027718	4.271952
C	-24.093438	-21.234425	3.548037
H	-26.887585	-19.633963	4.980781
H	-26.904121	-17.442624	3.857449
H	-27.075748	-17.305889	1.374337
H	-27.320656	-19.375951	0.039772
H	-27.529327	-21.814172	-0.082376

H	-28.018683	-24.211475	-0.204253
H	-28.386084	-26.387870	0.915804
H	-28.216494	-26.551137	3.397512
H	-27.598658	-24.575589	4.731176
H	-25.109505	-24.949188	5.039798
H	-24.790361	-27.064597	3.820719
H	-23.992952	-27.044872	1.458971
H	-23.434316	-24.896270	0.364461
H	-23.221775	-22.457211	0.454169
H	-22.738538	-20.060416	0.612656
H	-22.685652	-17.962654	1.925396
H	-23.479318	-17.956537	4.288462
H	-24.398588	-20.007905	5.293518
S	-26.030573	-22.299306	5.252771

An-SO_2-An *eclipsed* coordinates

C	-41.005675	13.793334	5.206778
C	-41.358185	15.017098	4.528424
C	-41.502490	16.264655	5.171933
C	-41.759665	17.419089	4.449165
C	-41.872117	17.379307	3.049269
C	-41.759469	16.173007	2.390091
C	-41.532562	14.969710	3.105203
C	-41.455508	13.738194	2.433663
C	-41.415938	12.521452	3.135001
C	-41.527710	11.285156	2.449650
C	-41.526226	10.089974	3.138151
C	-41.410290	10.094920	4.538336
C	-41.263338	11.285654	5.232417
C	-41.238640	12.525287	4.558624
C	-38.442900	13.915819	5.126761
C	-38.131034	12.673624	4.462081
C	-37.942220	11.445156	5.130185
C	-37.727495	10.271105	4.425333
C	-37.704403	10.271473	3.020551
C	-37.863317	11.458372	2.336040
C	-38.047784	12.681119	3.029915
C	-38.171341	13.893340	2.330391
C	-38.165250	15.129243	2.998699
C	-38.097053	16.345985	2.273826
C	-38.054551	17.559741	2.927747
C	-38.083156	17.593720	4.331973
C	-38.186519	16.422839	5.066558
C	-38.252379	15.165097	4.430240
H	-41.413110	16.319081	6.248748
H	-41.871042	18.362865	4.974871
H	-42.053819	18.292570	2.490429
H	-41.855444	16.123801	1.308268

H	-41.542448	13.720884	1.349297
H	-41.627637	11.298734	1.367148
H	-41.620921	9.150354	2.601969
H	-41.431723	9.157845	5.086927
H	-41.168841	11.266338	6.309978
H	-37.962288	11.421273	6.211620
H	-37.579571	9.342768	4.969229
H	-37.555262	9.343038	2.477300
H	-37.836586	11.476917	1.249338
H	-38.154850	13.880217	1.242610
H	-38.065567	16.302334	1.187961
H	-37.993367	18.484014	2.360885
H	-38.027828	18.545570	4.852007
H	-38.214324	16.472094	6.147020
O	-39.603042	12.638655	7.182376
O	-39.722484	15.125006	7.152395
S	-39.686477	13.871270	6.391785

Optimized MECP coordinates for the An-S-An dimer (in Angstroms).

An-S-An *elongated* coordinates

C	-5.001778	-0.098991	0.846047
C	-4.199171	0.956670	0.328002
C	-4.787420	2.104849	-0.195271
C	-2.771902	0.828498	0.348168
C	-4.020571	3.147235	-0.708460
C	-2.590492	3.052427	-0.706689
C	-1.988241	1.887481	-0.171938
C	4.765917	1.296822	-0.085047
C	4.725951	0.895838	2.341270
C	4.062318	1.165940	1.106207
C	4.028977	0.774538	3.499312
C	2.626104	1.306212	1.110544
C	4.130524	1.563820	-1.291781
C	2.051064	1.571349	-0.116891
C	1.934431	1.171589	2.352431
C	2.695341	1.713368	-1.330112
C	2.613135	0.915618	3.500400
H	2.073758	0.818401	4.428736
H	0.864247	1.281349	2.352191
S	-0.187946	1.749660	-0.147897
C	-2.223673	-0.364313	0.890265
C	-4.435153	-1.225697	1.358456
H	-2.579638	-2.250261	1.781772
C	-3.025325	-1.355271	1.378236
H	-1.154469	-0.476076	0.907366
H	5.839338	1.186116	-0.072880

H	4.542674	0.572925	4.425216
H	5.799941	0.792516	2.326807
H	-5.863332	2.188900	-0.203521
H	-6.074278	0.017118	0.820789
H	-5.051391	-2.020305	1.747169
C	4.230763	1.943255	-3.684728
C	4.862747	1.690774	-2.510725
C	2.815759	2.088442	-3.719045
C	2.073100	1.977674	-2.587237
H	4.795421	2.034635	-4.598364
H	2.328096	2.287735	-4.659672
H	1.002859	2.083798	-2.611796
H	5.935204	1.577484	-2.471505
C	-2.487282	5.245794	-1.742524
H	-1.902172	6.059425	-2.140280
C	-1.856189	4.144221	-1.241577
C	-3.900229	5.337067	-1.742463
H	-4.379825	6.217082	-2.139574
C	-4.642131	4.313207	-1.238227
H	-0.782438	4.088954	-1.242628
H	-5.719766	4.365458	-1.228041

References

1. J. B. Birks, Excimers, *Rep. Prog. Phys.*, 1975, **38**, 903.
